# A comprehensive study of Z-DNA density and its evolutionary implications in birds

**DOI:** 10.1186/s12864-024-11039-x

**Published:** 2024-11-21

**Authors:** Yu-Ren Wang, Shao-Ming Chang, Jinn-Jy Lin, Hsiao-Chian Chen, Lo-Tung Lee, Dien-Yu Tsai, Shih-Da Lee, Chung-Yu Lan, Chuang-Rung Chang, Chih-Feng Chen, Chen Siang Ng

**Affiliations:** 1 Institute of Molecular and Cellular Biology, National Tsing Hua University, Hsinchu, 300044 Taiwan; 2 National Center for High-performance Computing, National Applied Research Laboratories, Hsinchu, 300092 Taiwan; 3 Marine Research Station, Academia Sinica, Yilan, 262204 Taiwan; 4 Okinawa Institute of Science and Technology, Okinawa, 904-0495 Japan; 5 Department of Life Science, National Tsing Hua University, Hsinchu, 300044 Taiwan; 6 Institute of Biotechnology, National Tsing Hua University, Hsinchu, 300044 Taiwan; 7 Department of Medical Science, National Tsing Hua University, Hsinchu, 300044 Taiwan; 8 School of Medicine, National Tsing Hua University, Hsinchu, 300044 Taiwan; 9 Deparment of Animal Sciences, National Chung Hsing University, Taichung, 402202 Taiwan; 10 The iEGG and Animal Biotechnology Center, National Chung Hsing University, Taichung, 402202 Taiwan; 11 Bioresource Conservation Research Center, National Tsing Hua University, Hsinchu, 300044 Taiwan

## Abstract

**Background:**

Z-DNA, a left-handed helical form of DNA, plays a significant role in genomic stability and gene regulation. Its formation, associated with high GC content and repetitive sequences, is linked to genomic instability, potentially leading to large-scale deletions and contributing to phenotypic diversity and evolutionary adaptation.

**Results:**

In this study, we analyzed the density of Z-DNA-prone motifs of 154 avian genomes using the non-B DNA Motif Search Tool (nBMST). Our findings indicate a higher prevalence of Z-DNA motifs in promoter regions across all avian species compared to other genomic regions. A negative correlation was observed between Z-DNA density and developmental time in birds, suggesting that species with shorter developmental periods tend to have higher Z-DNA densities. This relationship implies that Z-DNA may influence the timing and regulation of development in avian species. Furthermore, Z-DNA density showed associations with traits such as body mass, egg mass, and genome size, highlighting the complex interactions between genome architecture and phenotypic characteristics. Gene Ontology (GO) analysis revealed that Z-DNA motifs are enriched in genes involved in nucleic acid binding, kinase activity, and translation regulation, suggesting a role in fine-tuning gene expression essential for cellular functions and responses to environmental changes. Additionally, the potential of Z-DNA to drive genomic instability and facilitate adaptive evolution underscores its importance in shaping phenotypic diversity.

**Conclusions:**

This study emphasizes the role of Z-DNA as a dynamic genomic element contributing to gene regulation, genomic stability, and phenotypic diversity in avian species. Future research should experimentally validate these associations and explore the molecular mechanisms by which Z-DNA influences avian biology.

**Supplementary Information:**

The online version contains supplementary material available at 10.1186/s12864-024-11039-x.

## Introduction

In Z-DNA, the sugar-phosphate backbone follows a zigzag pattern, hence the name “Z-DNA” [1–3]. This zigzag conformation results in a more compact and elongated structure than B-DNA [4, 5]. Z-DNA has attracted attention because of the frequent presence and conservation of Z-DNA-forming sequences among various eukaryotic species [6, 7]. Specific base-pairing sequences drive its unique structure and are typically stabilized under conditions such as high salt concentrations or the presence of certain cations [8, 9]. This conformation can form transiently under physiological conditions, especially in regions with alternating purine-pyrimidine sequences [10, 11].

Z-DNA formation is favored by sequences rich in alternating CG (cytosine-guanine) repeats [12]. This helical configuration arises due to the physical and chemical properties of these specific nucleotide pairings, which induce the DNA to adopt a left-handed spiral structure under certain conditions, such as negative supercoiling and physiological ionic strength. This alteration also readily occurs with alternating CA sequences on one strand and TG sequences on the complementary strand [13, 14]. The inherent instability of these sequences can create local torsional stress, further promoting the transition from the typical right-handed B-DNA to left-handed Z-DNA. Other sequences, including alternating purine-pyrimidine tracts such as (GA)_n_ or (GT)_n_ repeats, can also adopt this configuration under specific conditions. The propensity of these sequences to form Z-DNA is influenced by factors like sequence length, superhelical density, and the presence of stabilizing proteins [15].

Z-DNA plays a significant role in several fundamental biological processes, including transcription [16], epigenetics [17], DNA damage repair [18], genome stability [19, 20], genome evolution [21], recombination [22], RNA editing [23], and signal transduction [24]. Its ability to form in response to supercoiling and interact with various proteins underscores its multifaceted role in regulating and maintaining genomic functions [5].

Z-DNA formation induces genomic instability by promoting double-strand breaks (DSBs) [18, 20, 25]. These breaks constitute a critical form of DNA damage that can lead to severe genetic alterations if not correctly repaired. Z-DNA’s unique left-handed helical structure creates tension and torsional stress within the DNA molecule, making it more susceptible to breakage [18]. When DSBs occur, the cell’s repair mechanisms attempt to fix the damage, but these processes can be error-prone. For instance, microhomology-mediated end-joining (MMEJ) is a repair mechanism that aligns short homologous sequences flanking the breakpoints [26] but it is less accurate than homologous recombination, often resulting in deletions or insertions. This error-prone repair can lead to large-scale deletions, altering the genomic landscape significantly and making Z-DNA regions hotspots for genetic variation, thereby contributing to natural selection and adaptive evolution [21].

An example of Z-DNA-induced genomic instability driving phenotypic evolution is observed in sticklebacks, where the transition from marine to freshwater environments has led to the repeated loss of pelvic fins [27, 28]. This phenotypic change is primarily driven by deletions in a specific enhancer region of the *PITX1* gene, which is crucial for pelvic fin development [29]. The enhancer region in marine sticklebacks contains TG-dinucleotide repeats prone to forming Z-DNA, making this region particularly susceptible to double-strand breaks. These deletions silence *PITX1* expression in developing pelvic fins, resulting in their loss in freshwater populations [27, 28]. This process has occurred independently in different stickleback populations, demonstrating how Z-DNA can facilitate rapid and repeated evolutionary adaptations. This evolutionary convergence highlights the importance of genomic regions prone to Z-DNA formation in driving phenotypic diversity and adaptability in response to environmental pressures.

Similarly, variations in *PITX1* expression contribute to notable phenotypic traits like foot feathering in domestic birds, such as pigeons and chickens. A 44-kb deletion upstream of *PITX1* in pigeons [30] and a 17.7-kb deletion in chickens are associated with this phenotype [31, 32]. These deletions are selected traits in domestic breeds, highlighting the role of *PITX1* in phenotypic diversity. The regulation of *PITX1* involves complex genetic interactions [33]. Deletions disrupting conserved elements near the *PITX1* gene can significantly alter its expression. One such regulatory element is the pan-limb enhancer (*Pen)*, which interacts differently with *PITX1* in forelimbs and hindlimbs [34]. Structural mutations repositioning *Pen* near *PITX1* can cause ectopic expression, leading to homeotic transformations such as the arm-to-leg transformation observed in Liebenberg syndrome [35]. These regulatory mechanisms underscore the intricate control required for *PITX1* expression and its impact on limb development. The 17-kb deletion associated with foot feathering in chickens involved a 7-bp microhomology at the deletion junctions [31]. We propose that the formation of Z-DNA in these regulatory regions can contribute to the observed genomic instability and subsequent deletions.

Given its involvement in phenotypic variation and gene expression regulation, we speculate that the density of Z-DNA-forming sequences in the genome is a crucial factor influencing genomic stability, gene regulation, adaptive evolution, and phenotypic diversity in birds. High-density Z-DNA regions are prone to genomic instability, leading to increased mutation rates, double-strand breaks (DSBs), and large-scale deletions or rearrangements. These mutations and structural variations often occur in regulatory regions of the genome, such as enhancers and promoters, where they can significantly alter gene expression patterns. This genomic instability is a double-edged sword; while it can lead to deleterious mutations, it also provides a rich source of genetic variation essential for evolutionary processes.

Yeast artificial chromosomes (YAC) were widely provided as a good tool in chromosomal function and stability study, especially for non-B DNA-induced fragility [18, 36–39]. Shuttle vectors usually contain centromeres, telomeres, and specific selection markers, enabling them to replicate in bacteria and eukaryotic cells. Another feature of YACs is that the largest size of inserts could be approximately 100 kb [40]. All above allow YACs to mimic eukaryotic genomic DNA’s metabolic processes, including replication, damage, repair, and chromatinization.

Critical life-history traits such as developmental time, genome size, body mass, and egg mass are closely tied to an organism’s growth, reproduction, and survival, making them critical indicators of evolutionary fitness. For instance, developmental time is closely linked to species-specific life strategies [41, 42], where faster-developing species may be subject to different genomic pressures than those with more extended developmental periods. Similarly, genome size varies among avian species and is often associated with metabolic efficiency and adaptations to distinct ecological niches [43, 44]. Body and egg mass also reflect physiological and reproductive strategies shaped by evolutionary selection. We hypothesize that species with shorter developmental periods and higher metabolic demands may display higher Z-DNA densities, potentially influencing adaptive responses through genomic instability and variation.

To better understand the impact of Z-DNA density on avian biology, we explored its correlation with these critical life-history traits. By investigating the relationships between Z-DNA density and characteristics such as developmental time, genome size, body mass, and egg mass, we aim to uncover genomic regulatory patterns that shape species development and evolutionary trajectories. Understanding these correlations will shed light on how Z-DNA contributes to the evolution of avian species and the maintenance of phenotypic diversity. In this study, we applied phylogenetic linear regression models to analyze the correlation between Z-DNA density and various physiological traits, including adult body mass, egg mass, and developmental durations, providing a comprehensive view of Z-DNA’s role in avian biology. Additionally, we examined the density of Z-DNA-prone motifs in the promoter regions of avian genomes. This study is among the first to systematically investigate Z-DNA density in avian species, correlating it with developmental and phenotypic traits unique to birds.

## Materials and methods

### S1 nuclease test

Two web-based resources, “non-B DNA Motif Search Tool” (nBMST) (version 2.0) [45] and function “zdna: Predicting Z-DNA motif(s)” on R package “gquad” documentation (https://CRAN.R-project.org/package=gquad), were applied to predict possible “spots” that could form Z-DNA (Table S1) [46]. The Z-DNA search criteria are that one strand must contain alternating purine/pyrimidine sequences (such as GT and GC repeats). The S1 nuclease was used to identify sequences recognized as Z-DNA in the nBMST to determine Z-DNA formation in plasmids after synthesis. S1 nuclease specifically degrades single-stranded nucleic acids and cleaves at the junctions between right-handed and left-handed DNA segments [47]. It has also been utilized to detect the formation of supercoils [18, 48]. For the S1 nuclease assay, a mixture containing approximately 1000 ng of plasmid DNA, 0.1 µl of S1 nuclease, 6 µl of 5X reaction buffer, and an appropriate amount of water to a final volume of 30 µl was prepared. The mixture was incubated at room temperature for 30 min, and the reaction was terminated by adding 2 µl of 0.5 M EDTA, followed by heating at 70 °C for 10 min.

### YAC assay

A yeast artificial chromosome (YAC) was used to investigate the impact of Z-DNA sequences detected around the *Pen* region on genomic stability, following the procedures outlined by Polleys and Freudenreich (2020) [39]. In our experiment, the pRS415 plasmid—a YC-type shuttle vector with *LEU2* selection for *Saccharomyces cerevisiae*—was obtained from ATCC^®^, and Omics Bio synthesized the ZP1 and ZP4 sequences. The pRS415 vector was transformed into wild-type yeast strains (W303-1a) and plated on LEU- media for the fragility assay. All subsequent experiments were conducted at an incubation temperature of 30 °C. The yeast colonies with the transformed pRS415 plasmid were incubated in a YPD medium to induce the loss of *LEU2* for approximately 22 h. Afterward, 100 µl of the cultures were diluted to a specific concentration and plated on YPD plates. These were then replica plated on LEU-media after two days. The survival ratio of colonies between LEU- and YPD plates was calculated for each culture, with and without the Z-DNA insert. Additionally, amplification of the *LEU2* gene was performed to determine if the failure to grow on LEU- plates was due to the loss of the selection marker. We employed the maximum likelihood method using the fluctuation analysis calculator, FALCOR [43], to estimate the recombination rate.

### Genomic Z-DNA and short tandem repeat (STR) motifs search

The Non-B DNA Motif Search Tool (nBMST) (version 2.0) [45, 46, 49] was utilized to identify Z-DNA and short tandem repeat (STR) motifs using the computational resources of the National Center for High-performance Computing (NCHC). The genomes of all available avian species (154 species, Table S6) from the UCSC Genome Browser [50] were included in the search. The primary haplotype or the most recent version was used for species with multiple assembly versions.

### Z-DNA and STR motif density and ancestral state reconstruction

The phylogeny of the 154 avian species (Table S6) was obtained from BirdTree.org [51] using the Ericson all-species tree source to create 1000 trees. A consensus tree was generated using TreeAnnotator (version 1.10.4) [52] from these 1000 trees. Z-DNA and STR motif densities for each species were calculated by dividing the number of motifs by the genome size. The function fitContinuous from the package geiger (version 2.0.11) [53] was used to fit Z-DNA and STR motif densities to the “BM”, “OU”, and “EB” models on the consensus tree. The “OU” model was the best fit for both Z-DNA and STR motifs according to AICc values. The function fastAnc from the package phytools (version 1.5.1) [54] was then used to estimate the ancestral state of Z-DNA and STR densities, and the function contMap was used to plot the phylogeny tree with the ancestral states.

### Phylogenetic linear regression model

Data on several physiological traits, including adult body mass, egg mass, genome size, developmental duration, incubation duration, and fledging duration, were obtained from previous studies [55, 56]. These traits and adjusted measures, such as body mass-adjusted durations and egg mass-adjusted durations, were used to develop phylogenetic linear regression models incorporating Z-DNA and STR motif densities. Each pair of traits was analyzed across three categories: “all species”, “non-Passeriformes species”, and “Passeriformes species”. The treedata function was used to exclude species lacking trait data from the phylogenetic analysis. Three evolutionary models—“BM” (Brownian Motion), “OU” (Ornstein-Uhlenbeck), and “lambda”—were fitted using the phylolm function from the phylolm package (version 2.6.2) [57]. The best-fit model for each trait pair is provided in the supplementary table. Phylogenetic linear regression models were then constructed using the best-fit model for each trait pair, with 1,000 bootstraps to assess the robustness of the correlations. Two traits were considered correlated if the p-value was < 0.05 and the adjusted R-squared value was > 0.1.

### Z-DNA location analysis and gene ontology (GO)

To evaluate the distribution of Z-DNA motifs upstream of genes, the nBMST output TSV file was compared with the annotation GTF file to identify instances where the stop site of a Z-DNA motif appeared within 10-kb upstream of a start codon. The number of Z-DNA motifs per kbp within the 10 kbp upstream region of all start codons in the genome was then calculated. For this analysis, Augustus annotation files from the UCSC Genome Browser were used for each species, except for *Myiopsitta monachus* and *Apteryx mantelli.* Since an Augustus annotation file was unavailable for *Myiopsitta monachus*, the xenoRefGene annotation file was used instead. For *Apteryx mantelli*, assembly aptMan1 was used for motif searching, but no suitable annotation file was available for this assembly version.

To determine if the number of Z-DNA motifs within 1 kb of a start codon is significantly higher than in other regions, we first applied the Lilliefors test to assess whether the Z-DNA counts in each region followed a normal distribution. This was done using the lillie.test() function from the R package nortest (version 1.0.4) (https://cran.r-project.org/web/packages/nortest/index.html). Since the data did not follow a normal distribution, we proceeded with the Friedman test to examine whether the Z-DNA counts across different regions were significantly different. This was performed using the friedman.test() function in R (version 4.4.1). Following the Friedman test, Nemenyi’s all-pairs comparisons test was used to assess pairwise differences in Z-DNA counts between regions. This was implemented using the frdAllPairsNemenyiTest() function from the PMCMRplus package (version 1.9.12) (https://cran.r-project.org/web/packages/PMCMRplus/index.html).

To determine the functions of genes with the highest number of Z-DNA motifs in their upstream regions, the nBMST output TSV file was again compared with the annotation GTF file to check if the stop site of a Z-DNA motif appeared within 10 kb upstream of a gene’s start codon. The number of Z-DNA motifs within this 10-kb upstream region for all genes was then calculated. NCBI RefSeq annotation files from the UCSC Genome Browser were used for functional gene cluster analysis. The top 5% of genes with the highest Z-DNA motifs in each species were selected for functional clustering using the Database for Annotation, Visualization, and Integrated Discovery (DAVID) [58, 59]. Genes with the same number of Z-DNA motifs within their 10-kb upstream region as those in the fifth percentile were also included. Only 74 species were included in this analysis, as ncbiRefSeq annotation files were available for 88 species, and DAVID could not recognize the gene IDs of 14 species.

### Data availability

All raw data were deposited in the figshare with DOI nos. 10.6084/m9.figshare.26925115 and 10.6084/m9.figshare.26924593.

## Results

### Z-DNA motifs prediction and structure determination and fragility assay

**Fig. 1:**
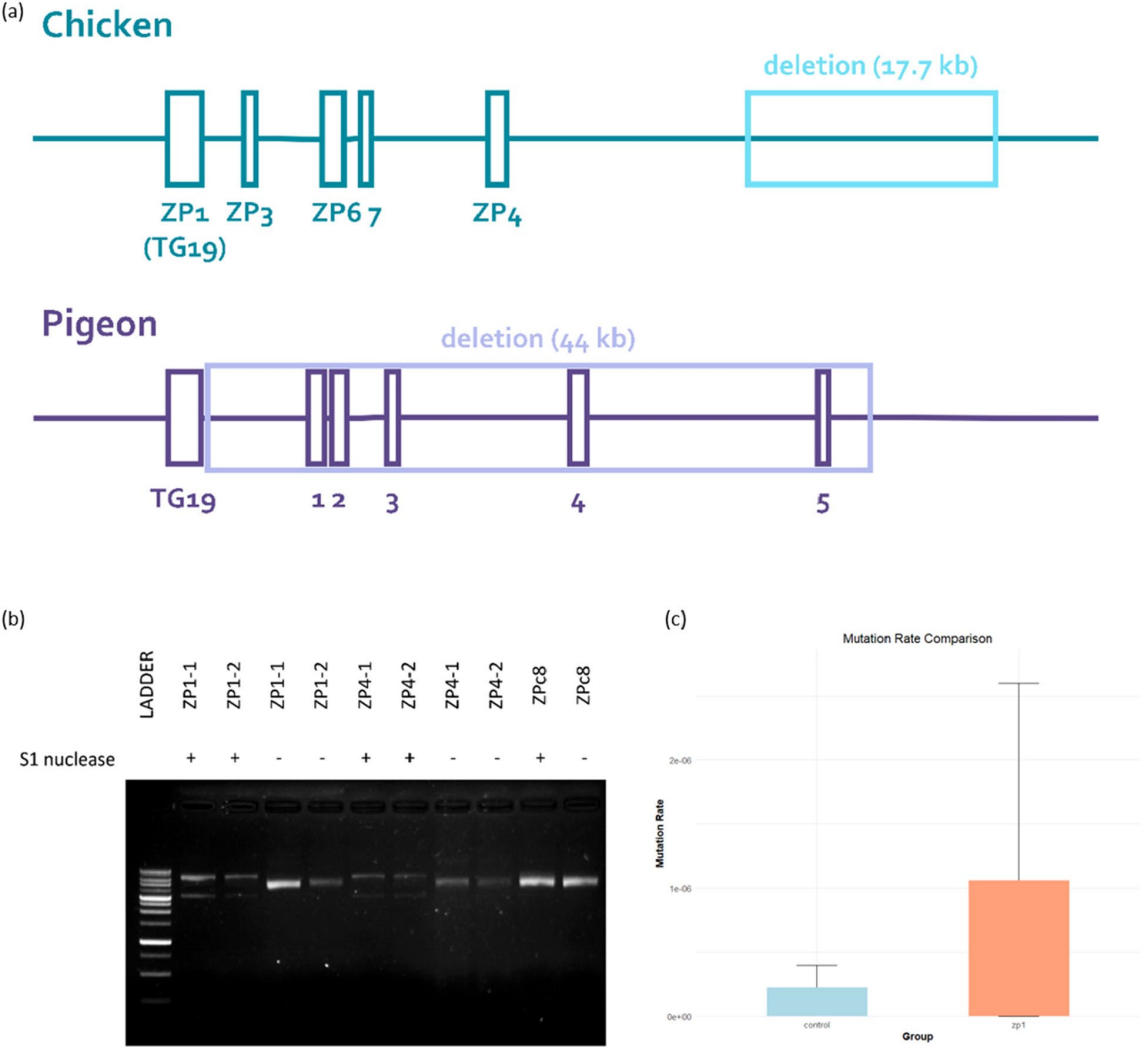
Distribution of Z-DNA motifs and structural analysis of supercoiled DNA. **a** Distribution of Z-DNA motifs in the Pen region of chickens and pigeons (not to scale). The analyzed regions correspond to chromosome 13 positions 16.05–16.11 Mb in chickens and scaffold 79 positions 6.715–6.775 Mb in pigeons. **b** S1 nuclease digestion assay of pUC57 vectors with and without Z-DNA insertions. Vectors containing the ZP1 and ZP4 motifs were susceptible to cleavage by S1 nuclease, indicating the presence of supercoiled structures associated with these motifs (original gel image shown in Fig. S11). **c** Fragility data of *S. cerevisiae* with the fragile sequence ZP1 integrated between the telomere seed sequence (G_4_T_4_)_13_ and URA3 markers. YAC assays demonstrate that 5-FOA resistance increases in the presence of ZP1

Understanding the distribution and density of Z-DNA motifs within key regulatory regions, such as the *Pen* region, provides valuable insights into potential gene regulation mechanisms and evolutionary pressures across species. The results of Z-DNA prediction on the *Pen* region are shown in Fig. 1a, with detailed information on each motif provided in Table S1. In chickens, five motifs were detected on chromosome 13 between 10.11 and 16.05 Mb, while in pigeons, six motifs were identified on scaffold 79 between 6.715 and 6.775 Mb. The average Z-DNA density in the *Pen* region is approximately one motif per 10 kb, about three times higher than the Z-DNA density across the entire genome in both chickens and pigeons. This suggests that the Z-DNA density in the *Pen* region is higher than in other genomic regions. Compared to pigeons, the distribution of Z-DNA motifs on the *PITX1* enhancers in chickens is more clustered and uneven, although the average Z-DNA density is similar in both species. Notably, both species’ most extended predicted motifs are identical in length, repeat type, and composition. In chickens and pigeons, the most extended motifs in the *Pen* region, ZP1 and TG19, respectively, consist of 19 TG repeats.

Repetitive sequences like TG repeats have been implicated in genomic instability and deletions, which can have functional consequences on gene regulation and phenotypic traits. Stickleback fish’s upstream regions of the Pel gene contain a TG20 repeat, which may be similar to the TG19 repeats found in chickens and pigeons [28]. These findings suggest that the *Pen* region could be considered a fragility site compared to the rest of the genome, primarily due to the TG19 repeat. Furthermore, the differences in the distribution and composition of each motif between chickens and pigeons could explain the variation in deletion scales in the *Pen* region.

Investigating the structural properties of Z-DNA sequences, such as ZP1 and ZP4, and their impact on chromosomal stability provides crucial insights into the role of Z-DNA in genome integrity. During the structure determination, ZP1 and ZP4 were cleaved when reacted with S1 nuclease (Fig. 1b), indicating Z-DNA formation in vitro. To test the effect of the ZP1 and ZP4 sequences on chromosome stability in vivo, we measured the rate of DNA double-strand breaks in yeast artificial chromosomes (YACs) (Fig. 1c, S1 and S2). Z-DNA-forming sequences were positioned on the right arm of a YAC upstream of the *URA3* gene. The left arm of the YAC contains the *LEU2* gene, essential for maintaining the YAC. If Z-DNA induces breakage, it will result in the loss of the right arm of the YAC, including the *URA3* gene, leading to resistance to 5-Fluoroorotic acid (FOA) (FOA resistance, FOA^R^). Thus, the rate of FOA^R^ cell generation was measured to monitor the breakage rate. A ZP1, ZP4, or a control B-DNA-forming sequence was inserted into the YAC adjacent to the *URA3* gene.

By conducting fragility assays, we aim to assess the capacity of Z-DNA-forming sequences to induce genomic breakage, even without exogenous stress factors, which may highlight their inherent instability and potential to cause gene loss or rearrangements under normal cellular conditions. In the fragility assay, both ZP1 and ZP4 sequences contributed to the loss of *LEU2* (Fig. 1c and S2). The highest rate of *LEU2* loss was observed with both ZP1 and ZP4 (Sign test, *P* = 0.02939) in the absence of exogenous DNA-damaging factors, with ZP4 also showing a marginal loss rate (Sign test, *P* = 0.12842).

### The evolution of Z-DNA density in bird

**Fig. 2:**
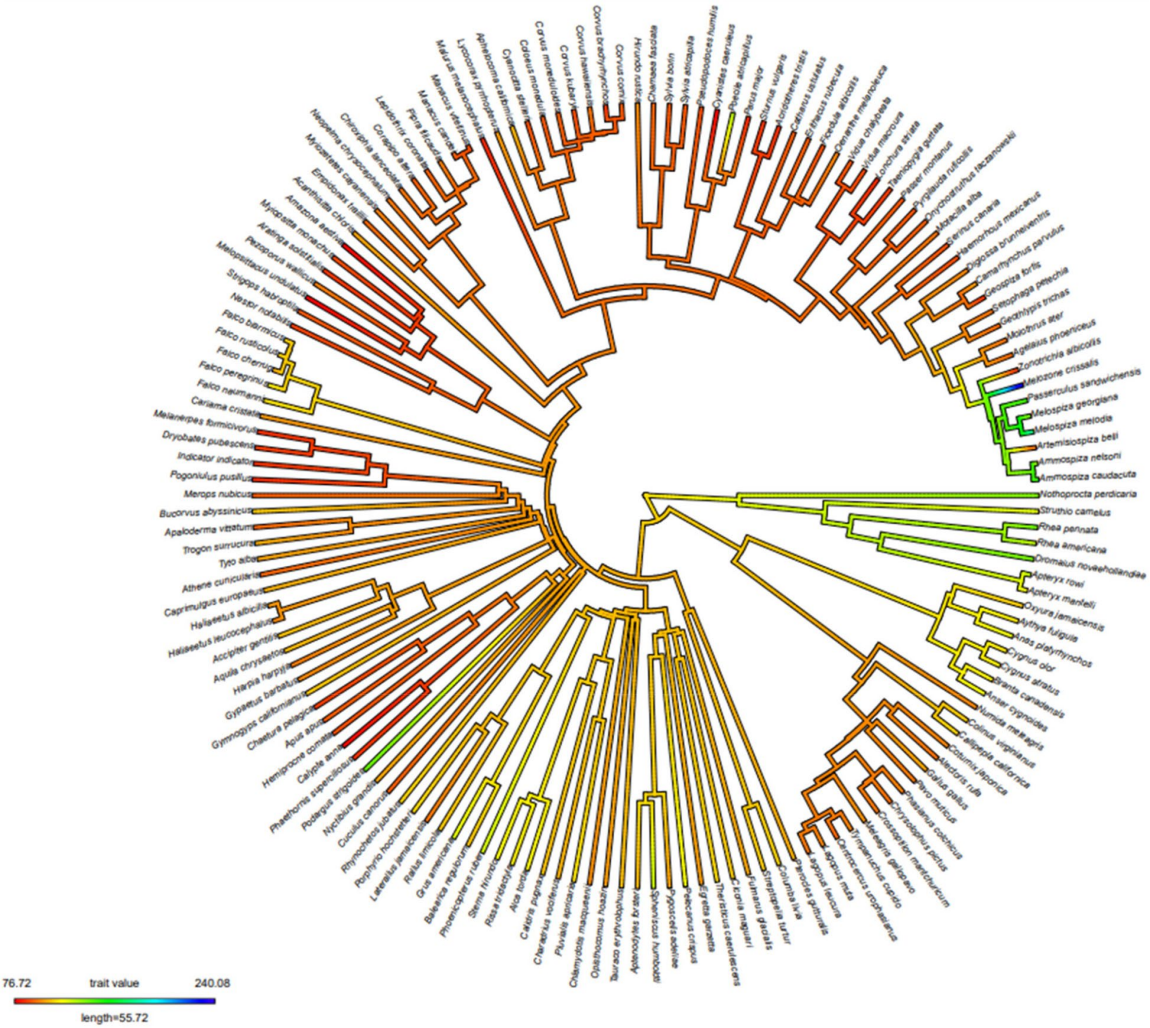
Ancestral state reconstruction of Z-DNA density across 154 avian species. The phylogenetic tree illustrates the evolutionary relationships among the avian species, with branch colors representing the estimated Z-DNA density. The color gradient from green to red indicates varying Z-DNA densities, ranging from low (red) to high (blue) values, with specific density values noted by the color scale at the bottom (76.72 to 240.08 motifs per Mb)

Investigating the genome-wide distribution of Z-DNA motifs and short tandem repeats (STRs) across avian species provides valuable insights into how these structural features contribute to genetic diversity and evolution. By comparing Z-DNA densities and STR densities, we aim to uncover patterns of genomic organization and evolutionary changes within and across bird lineages. The whole genome Z-DNA densities and STRs densities of 154 avian species (Table S6 and S7) were conducted to ancestral state reconstruction analysis. Our phylogenetic tree analysis reveals substantial interspecific variation in Z-DNA density among the studied bird species, with values ranging from 76.72 to 240.08 motifs per megabase (Mb). Palaeognathae birds exhibit a higher density of Z-DNA than Neognathae birds (Fig. 2). Within Neognathae, both Anseriformes and the basal lineages of Neoaves show a higher Z-DNA density. In general, Z-DNA density is lower in more derived lineages within Neognathae. Passerine birds typically have relatively low Z-DNA density, except for species in the Passerellidae family, where an expansion in Z-DNA density may have occurred in their common ancestor. Furthermore, Z-DNA expansion has occurred sporadically in Neognathae birds, with notable increases in certain passerine and non-passerine species. The ancestor of the genus *Falco* may also have experienced a rise in Z-DNA density.

Short Tandem Repeats (STRs) are DNA sequences where a short sequence of base pairs is repeated consecutively. The repeated units typically consist of 2–6 base pairs, and these repeats can occur a variable number of times within a specific region. Similarly, analysis of STR density reveals significant interspecific variation, ranging from 479.06 to 1,353.89 motifs per Mb (Fig. S3). The phylogenetic changes in STRs differ markedly from those observed in Z-DNA, suggesting that these two genomic elements have distinct evolutionary trajectories. Unlike Z-DNA, STR density is not exceptionally high in Palaeognathae birds and has decreased in Psittaciformes, indicating that the evolutionary pattern of Z-DNA density may be unique.

### Developmental time predicts Z-DNA density in birds

To explore the evolutionary relationships between Z-DNA density and critical life-history traits, we performed a PGLS analysis across 154 avian species, integrating data on both non-passerine and passerine birds. Given the variable availability of trait data across species, the sample sizes for each analysis differed accordingly. The results of the PGLS model confirmed that Z-DNA density in birds is significantly and negatively correlated with developmental time (Table S2, Fig. 4). A lambda or an OU model, which transforms the phylogeny into a covariance matrix, provided the best fit for examining the relationships among these variables (Table S2).

**Fig. 3:**
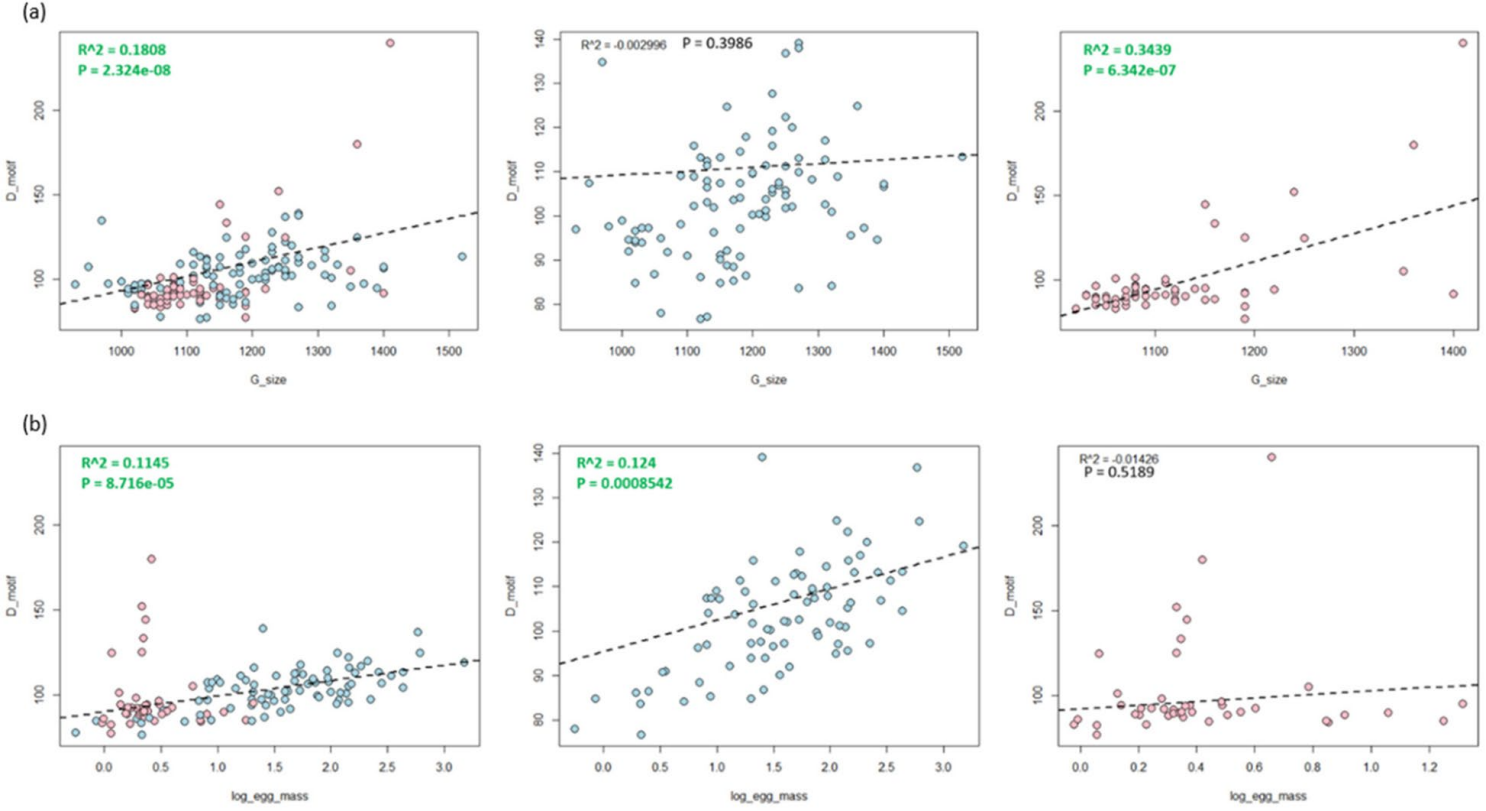
Phylogenetic linear regression analysis showing the relationship between Z-DNA density and (**a**) genome size and (**b**) egg mass in avian species. Blue dots represent non-Passeriformes species, while pink dots represent Passeriformes species. Values are green for moderate correlations, while weak correlations are red

All 154 species were included in the PGLS analysis of Z-DNA density and genome size. An analysis of the relationship between genome size and Z-DNA density across different bird groups reveals distinct patterns (Fig. 3a). In the overall group of bird species, there is a weak positive correlation (*R*² = 0.1808, *p* = 2.324 × 10^−8^), suggesting a slight increase in Z-DNA density with larger genome sizes. For non-passerine birds, there is no significant correlation (*R*² = −0.002996, *p* = 0.3986), indicating no association between genome size and Z-DNA density. In contrast, passerine birds show a moderate positive correlation (*R*² = 0.3439, *p* = 6.342 × 10^−7^), indicating a more pronounced increase in Z-DNA density with increasing genome size. These findings suggest that different evolutionary pressures or functional roles may influence Z-DNA density between passerines and non-passerines, with passerines showing a stronger association.

All 154 species were included in the PGLS analysis of STR density and genome size. Conversely, the analysis reveals a minimal correlation between genome size and STR density across all groups (Fig. S5a). The passerines show a weak positive correlation (*R*² = 0.08513, *p* = 0.0142235), while overall birds and non-passerines exhibit no significant relationships (*R*^²^ = 0.01382, *p* = 0.07821 and *R*^²^ = −0.007905, *p* = 0.609420, respectively). This indicates that STR density is not substantially influenced by genome size, highlighting the need to consider other factors in determining STR density.

There were 153 species, including 95 non-passerine birds and 58 passerine birds, in the PGLS analysis of Z-DNA density and body mass. Body mass shows minimal influence when examining the relationship between body mass and Z-DNA density across bird groups (Fig. S4a). The overall birds display a weak positive correlation (*R*² = 0.09769, *p* = 4.957 × 10^−5^), while non-passerines and passerines exhibit no significant relationships (*R*² = 0.04387, *p* = 0.02339 and *R*² = −0.01746, *p* = 0.8833, respectively). These results suggest that body mass is not a significant determinant of Z-DNA density in birds.

There were 121 species, including 79 non-passerine birds and 42 passerine birds, in the PGLS analysis of Z-DNA density and egg mass. The relationship between egg mass and Z-DNA density also varies among bird groups (Fig. 3b). For the overall bird group, there is a positive correlation (*R*² = 0.1145, *p* = 8.716 × 10^−5^), with non-passerines showing a more pronounced correlation (*R*² = 0.124, *p* = 0.0008542) and passerines showing no significant correlation (*R*² = −0.01426, *p* = 0.5189). This suggests that egg mass may have an influence on Z-DNA density, particularly in non-passerines, potentially due to specific evolutionary factors.

There were 127 species, including 81 non-passerine birds and 46 passerine birds, in the PGLS analysis of Z-DNA density and developmental duration (Fig. S4d). Across all birds, the correlation is weak and negligible (*R*² = 0.03286, *p* = 0.02322), with non-passerines and passerines (*R*² = 0.03159, *p* = 0.06108 and *R*² = 0.03639, *p* = 0.1075 respectively) showing a similar trend. These findings suggest that developmental duration is not a reliable predictor of Z-DNA density in birds.

**Fig. 4:**
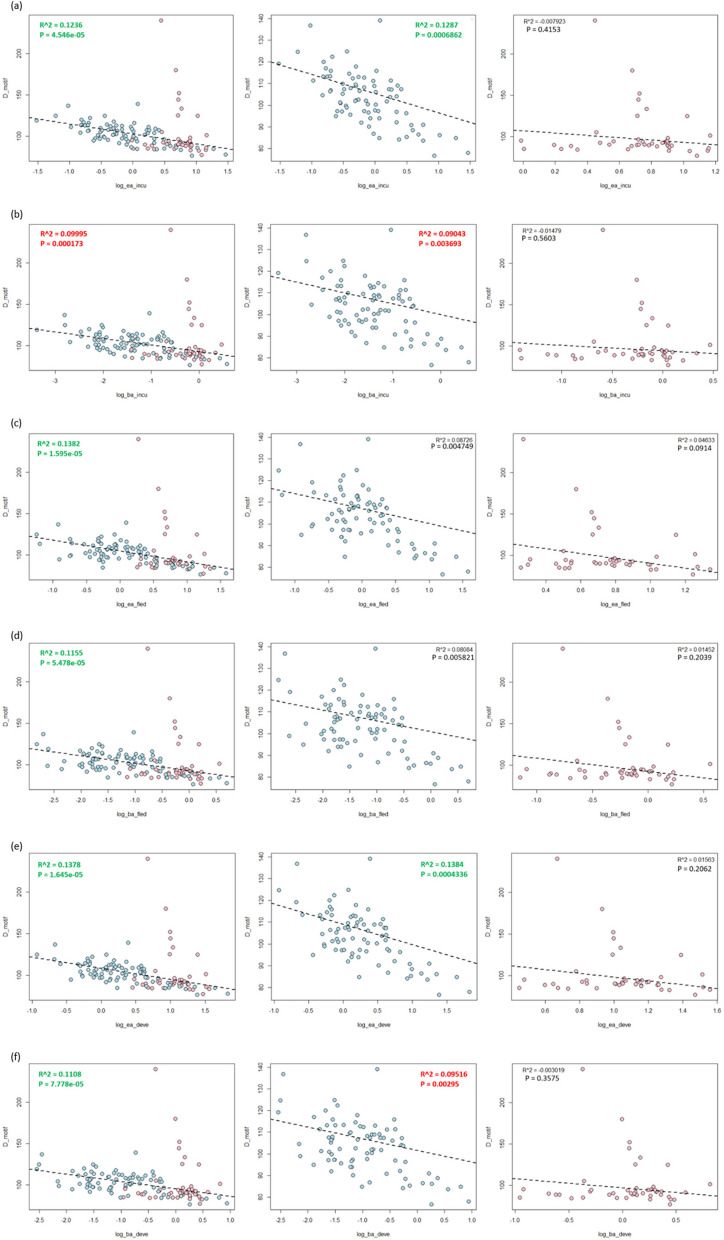
Phylogenetic linear regression between Z-DNA and adjusted developmental periods. **a** Egg mass adjusted incubation time. **b** Adult body mass adjusted incubation time. **c** Egg mass adjusted fledging time. **d** Adult body mass adjusted fledging time. **e** Egg mass adjusted developmental time. **f** Adult body mass adjusted developmental time. The blue spots indicate the non-Passeriformes species and the pink spots indicate the Passeriformes species. Each column within the rows represents different bird groups: all birds (left), non-Passeriformes (middle), and Passeriformes (right). Blue dots indicate non-Passeriformes species, while pink dots represent Passeriformes species. The *R*² values and *p*-values provided in each plot indicate the strength and significance of the relationships, respectively. The dashed lines represent the regression lines, illustrating the trend of the relationships between Z-DNA density and the corresponding traits. Values are green for moderate correlations, while weak correlations are red

There were 127 species, including 81 non-passerine birds and 46 passerine birds, in the PGLS analysis of Z-DNA density and body mass-adjusted developmental time. The analysis shows varying correlations between body mass-adjusted developmental time and Z-DNA density (Fig. 4f). The overall birds have a negative correlation (*R*² = 0.1108, *p* = 7.778 × 10^−5^), non-passerines show a weak negative correlation (*R*² = 0.09516, *p* = 0.00295), and passerines show no substantial correlation (*R*² = −0.003019, *p* = 0.3575). This suggests that body mass-adjusted developmental time does not significantly influence Z-DNA density in passerines. There were 121 species, including 79 non-passerine birds and 42 passerine birds, in the PGLS analysis of Z-DNA density and egg mass-adjusted developmental time. Similarly, the relationship between egg mass-adjusted developmental time and Z-DNA density show some correlations (Fig. 4e). Overall birds have a negative correlation (*R*² = 0.1378, *p* = 1.645 × 10^−5^), non-passerines also have a negative correlation (*R*² = 0.1384, *p* = 0.0004336), and passerines have no significant correlation (*R*² = 0.01563, *p* = 0.2062). These findings suggest an impact of egg mass-adjusted developmental time on Z-DNA density.

There were 127 species, including 81 non-passerine birds and 46 passerine birds, in the PGLS analysis of Z-DNA density and incubation duration. Incubation duration correlates poorly with Z-DNA density across bird groups (Fig. S4b). Overall birds and non-passerines exhibit weak positive correlations (*R*² = 0.06223, *p* = 0.002711 and *R*² = 0.04846, *p* = 0.02706, respectively), while passerines show no correlation (*R*² = −0.01487, *p* = 0.56242). This indicates that incubation duration has a negligible effect on Z-DNA density.

There were 127 species, including 81 non-passerine birds and 46 passerine birds, in the PGLS analysis of Z-DNA density and body mass-adjusted incubation duration. Correlations between body mass-adjusted incubation duration and Z-DNA density are weak (Fig. 4b). The passerines show negligible correlations (*R*² = −0.01479, *p* = 0.5603). At the same time, overall birds and non-passerines exhibit a slightly stronger negative correlation (*R*² = 0.09995, *p* = 0.000173 and *R*² = 0.09043, *p* = 0.003693, respectively). The influence of body mass-adjusted incubation duration on Z-DNA density appears minimal (Fig. 4b). There were 121 species, including 79 non-passerine birds and 42 passerine birds, in the PGLS analysis of Z-DNA density and egg mass-adjusted incubation duration. Egg mass-adjusted incubation duration shows negative correlations with Z-DNA density across bird groups, suggesting a minimal impact on Z-DNA density (Fig. 4a). Stronger correlations are observed in overall birds and non-passerines (*R*² = 0.1236, *p* = 4.546 × 10^−5^ and *R*² = 0.1287, *p* = 0.0006862, respectively). In contrast, the passerines show negligible correlations (*R*² = −0.007923, *p* = 0.4153).

There were 127 species, including 81 non-passerine birds and 46 passerine birds, in the PGLS analysis of Z-DNA density and fledging duration. Fledging duration shows varying correlations with Z-DNA density (Fig. S4c). Overall birds and non-passerines exhibit no significant correlations (*R*² = 0.01379, *p* = 0.099 and *R*² = 0.02334, *p* = 0.09187, respectively), while the passerines show weak negative correlations (*R*² = 0.0731, *p* = 0.03855). The data suggest that Z-DNA density slightly decreases with longer fledging durations in passerines.

There were 127 species, including 81 non-passerine birds and 46 passerine birds, in the PGLS analysis of Z-DNA density and body mass-adjusted fledging duration. The analysis shows varying correlations between body mass-adjusted fledging duration and Z-DNA density (Fig. 4d). Overall birds have a negative correlation (*R*² = 0.1155, *p* = 5.478 × 10^−5^), non-passerines show a weak negative correlation (*R*² = 0.08084, *p* = 0.005821), and the passerines show no significant correlation (*R*² = 0.01452, *p* = 0.2039). There were 121 species, including 79 non-passerine birds and 42 passerine birds, in the PGLS analysis of Z-DNA density and egg mass-adjusted fledging duration. Similar correlations are shown between egg mass-adjusted fledging duration and Z-DNA density (Fig. 4c). Overall birds have a negative correlation (*R*² = 0.1382, *p* = 1.595 × 10^−5^), non-passerines show a weak negative correlation (*R*² = 0.08726, *p* = 0.004749), and passerines show no significant correlation (*R*² = 0.04633, *p* = 0.0914).

Our analysis found no correlation between STR density or genome size and the phenotypes investigated. While significant relationships were identified between Z-DNA density and developmental time and varying correlations with factors like egg mass and body mass across different bird groups, STR density showed no meaningful association with genome size or the phenotypes studied. These results highlight the absence of any substantial correlation between STR density or genome size and the phenotypic traits examined (Table S3-4, Figures S5-9).

**Fig. 5:**
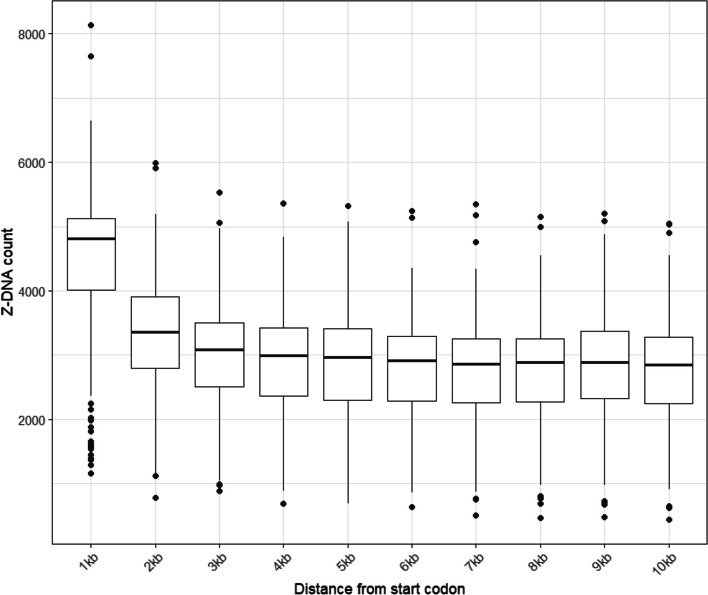
Z-DNA number in different regions upstream from a start codon. Boxplots show the first quartile, median and third quartile of Z-DNA number in each region. Black dots show the outlier of each region

### Positional frequency of Z-DNA in birds

To understand promoter-Z-DNA associations comprehensively, we measured the positional frequency in 153 avian species, defined as the total number of Z-DNA motifs at each nucleotide position within 10 kb upstream of the start codon. This method allows us to identify regions where Z-DNA motifs are concentrated, providing insights into their potential regulatory roles. Our analysis shows that the regions immediately upstream of the start codon, particularly within the first 1 kb, often have a higher concentration of Z-DNA motifs than the remaining 9 kb upstream (Fig. S10 and supplementary files). Friedman test showed a significant different of Z-DNA number between each region (Friedman chi-squared = 1066.1, d.f. = 10, *p*-value < 2.2 × −10^−16^). Pairwise *p*-value between the first 1 kb and other regions from Nemenyi’s all-pairs comparisons tests also showed that the Z-DNA number within 1 kb from start codon was significantly higher than other regions (Fig. 5 and table S8). This suggests that proximal promoter regions are particularly enriched with Z-DNA motifs, which could have important implications for transcriptional regulation.

Our Gene Ontology (GO) analysis of 74 avian species revealed a significant enrichment of genes associated with DNA or RNA binding activities among the top 5% Z-DNA-rich promoter regions (see supplementary files). This suggests that Z-DNA motifs may play critical roles in regulating genes involved in nucleic acid interactions, such as transcription factors, RNA polymerases, and other proteins that bind directly to DNA or RNA to control gene expression and RNA processing. Additionally, genes involved in kinase activity were significantly enriched in Z-DNA-rich regions (see supplementary files). Kinases are essential enzymes that regulate various cellular processes through phosphorylation, including signal transduction pathways, cell cycle regulation, and metabolic control. Z-DNA motifs near these genes suggest a potential regulatory mechanism whereby Z-DNA formation could influence kinase transcription, affecting numerous cellular functions and responses to environmental stimuli.

Another prominent category identified in the GO analysis included genes related to translation regulation (see supplementary files). These genes are involved in protein synthesis, encoding ribosomal proteins, and translation initiation and elongation factors. The enrichment of Z-DNA motifs in their promoter regions suggests that Z-DNA may play a role in fine-tuning the expression of genes critical for maintaining translational fidelity and efficiency, which is crucial for cellular growth and adaptation.

## Discussion

### Z-DNA density in avian genomes

Birds are notable for having the smallest genomes among amniotes, with relatively slight variation in genome size across different species [60, 61]. This characteristic distinguishes them from other groups, such as reptiles and mammals. The significant reduction in genome size and transposable element density in birds began after their evolutionary divergence from crocodilians [62]. This reduction likely occurred before the evolution of flight, suggesting that a smaller genome may have provided adaptive advantages, possibly related to increased metabolic efficiency and optimized cellular function during the development of flight [63]. Since the common ancestor of modern birds, genome size has decreased, although slower.

In addition to having small genomes, birds exhibit low densities of Z-DNA compared to non-avian reptiles and mammals. Z-DNA is a left-handed helical form of DNA that can form under physiological conditions and is often associated with transcriptionally active regions of the genome [5, 64, 65]. Despite the overall low density of Z-DNA in birds, there is considerable variation in Z-DNA density among different bird species. This variation suggests that other bird species may have evolved distinct regulatory mechanisms and genome organization strategies involving Z-DNA. The presence and density of Z-DNA could influence gene expression patterns and genomic stability, contributing to the diverse phenotypic traits observed among bird species.

Birds’ small genome size and low Z-DNA density may be linked to their unique physiological and ecological adaptations. A compact genome may reduce the energy and time required for DNA replication and transcription, crucial for sustaining high metabolic activities, such as those needed for flight. Additionally, the variation in Z-DNA density among bird species could reflect adaptive responses to different environmental pressures and lifestyle requirements. The evolutionary trajectory of genome size reduction and Z-DNA’s low but variable density in birds underscores the complex relationship between genomic architecture and avian adaptive strategies. Understanding these dynamics provides deeper insights into avian species’ molecular evolution and functional genomics.

Our findings suggest that Z-DNA density in birds is influenced by several factors, including developmental time, genome size, and egg mass, but not significantly by body mass. The varying correlations among bird groups indicate that Z-DNA may play different regulatory roles depending on specific evolutionary pressures and ecological adaptations. Understanding the dynamics of Z-DNA density in birds offers valuable insights into avian species’ molecular evolution and functional genomics. Future research should experimentally validate these associations and explore the underlying mechanisms driving these correlations. This could involve studying specific genes and regulatory pathways influenced by Z-DNA and examining how changes in Z-DNA density impact phenotypic diversity and ecological adaptation. By elucidating the roles of Z-DNA in gene regulation, we can better understand the evolutionary pressures shaping avian genomes and the potential impact of Z-DNA on bird biology and evolution.

In the phylogeny presented in Fig. 2, a distinct cluster of passerine birds exhibits a higher density of Z-DNA motifs. Notably, species such as *Ammospiza caudacuta* (Saltmarsh Sparrow), *Ammospiza nelsoni* (Nelson’s Sparrow), *Melospiza georgiana* (Swamp Sparrow), *Melospiza melodia* (Song Sparrow), and *Passerculus sandwichensis* (Savannah Sparrow), which are part of this cluster, are predominantly adapted to marshy or coastal habitats [66]. These environments subject the species to unique ecological pressures, including fluctuating tidal levels, high predation risk, and the need for precise timing in breeding cycles to avoid environmental hazards. Such factors likely drive specific genomic adaptations, including increased Z-DNA density in regulatory regions.

For instance, saltmarsh species like the Saltmarsh Sparrow have evolved mechanisms to withstand regular flooding, which may impose selective pressures on their genomes. These pressures could influence genomic traits such as Z-DNA motif distribution, gene regulation, and genome size, facilitating key developmental and metabolic processes needed to thrive in these challenging environments. Conversely, *Artemisiospiza belli* (Bell’s Sparrow), which inhabits dry, open landscapes [66], exhibits a lower Z-DNA density, possibly reflecting different ecological and evolutionary demands compared to the marsh-adapted species. *Melozone crissalis* (California Towhee) is an exception within that cluster, which exhibits a higher Z-DNA density despite living in drier, shrub-dominated landscapes [66]. This suggests that the California Towhee may have inherited a pre-existing increase in Z-DNA density from their common ancestor, evolving unique genomic adaptations that distinguish it from other desert species regarding Z-DNA density.

### Z-DNA in gene regulation

The elevated frequency of Z-DNA motifs within the 1 kb region upstream of the start codon underscores the significance of these structures in transcriptional regulation [49, 67]. Z-DNA formation in these regions can affect chromatin accessibility and the binding affinity of transcription factors [68–70]. The conformational changes induced by Z-DNA can alter the interaction between DNA and the transcriptional machinery, potentially enhancing or repressing gene expression [5, 71–73].

The concentration of Z-DNA motifs in proximal promoter regions suggests these areas may serve as regulatory hotspots. Z-DNA could facilitate the formation of specific chromatin structures or interact with Z-DNA-binding proteins, such as ADAR1 and ZBP1, which are involved in transcriptional regulation [74]. These interactions can act as molecular switches, turning genes on or off in response to various signals.

The preferential localization of Z-DNA motifs in critical regulatory regions highlights their potential role in evolutionary processes. Regions with high Z-DNA density may be under positive selection, driving adaptive changes in gene regulation. Variations in Z-DNA motif density and distribution can result in distinct gene expression patterns, contributing to the phenotypic diversity observed among different species [21]. Comparing the positional frequency of Z-DNA motifs across species could shed light on the evolutionary conservation of these regulatory elements. Species facing similar environmental pressures might exhibit convergent evolution in the density and positioning of Z-DNA motifs in promoter regions, reflecting shared adaptive strategies.

Enriching Z-DNA motifs in genes involved in fundamental processes, such as nucleic acid binding, kinase activity, and translation regulation, also suggests an evolutionary advantage. Genes under robust regulatory control may benefit from the added regulatory complexity provided by Z-DNA motifs [49, 67, 75, 76]. This complexity could allow for rapid adaptation to changing environments, conferring a selective advantage to organisms with such regulatory architectures.

Z-DNA structures are also implicated in various diseases, including cancer, neurological disorders, and genetic diseases [5, 20, 77, 78]. The formation of these structures can lead to genomic instability, mutations, and altered gene expression, contributing to disease pathogenesis. Understanding the roles of Z-DNA structures in health and disease could provide insights into potential therapeutic targets [5, 77, 79]. Drugs and small molecules explicitly targeting these structures are being explored for their potential in treating diseases associated with genomic instability and aberrant gene regulation.

### Z-DNA and phenotypic diversity

Z-DNA-induced deletions in regulatory regions have been implicated in the repeated loss of pelvic fins in stickleback populations, illustrating how Z-DNA contributes to phenotypic diversity and adaptation [28]. However, the role of Z-DNA in promoting phenotypic diversity is not limited to sticklebacks. Studies have shown that Z-DNA-forming sequences are often associated with large-scale deletions in mammalian cells [18]. These deletions can lead to significant phenotypic changes by removing or altering regulatory elements and coding regions. Z-DNA-induced genomic instability may thus be a crucial mechanism contributing to genetic diversity and the evolution of new traits in natural populations.

The impact of Z-DNA-induced genomic instability extends beyond mammals [21]. It has also been implicated in the evolution of various traits in other vertebrates. These large-scale deletions and the resulting genetic variations underscore the widespread influence of Z-DNA on evolutionary processes. Z-DNA-prone regions often serve as hotspots for genetic variation, driving adaptive evolution and the emergence of new phenotypic traits.

Z-DNA’s role as a critical driver of phenotypic diversity is rooted in its ability to induce genomic instability, facilitating large-scale deletions that can profoundly affect gene expression [80, 81]. By removing or altering regulatory elements and coding regions, Z-DNA-induced deletions can lead to significant changes in the genetic landscape, promoting the development of new traits and enhancing genetic diversity within populations [21].

The recurrent involvement of Z-DNA-prone regions in adaptive evolution underscores their importance in the evolutionary dynamics of animals. These regions are often associated with critical genetic changes that enable species to adapt to new environments or conditions. By serving as focal points for genetic variation, Z-DNA regions contribute to the evolutionary plasticity of organisms, allowing them to develop novel traits and enhance their survival and reproductive success.

Understanding the role of Z-DNA in phenotypic diversity provides valuable insights into the molecular mechanisms underlying evolution. It highlights the dynamic nature of genomes and the complex processes that drive genetic variation and evolutionary change. By studying Z-DNA and its effects on genomic stability, we can better understand how genetic diversity arises and how new traits evolve in natural populations.

### Other Non-B DNA

Non-B DNA structures, such as G-quadruplexes, cruciforms, and triplex DNA, play significant roles in genomic regulation, stability, and evolutionary processes [21, 77, 82, 83]. These structures interact extensively with the epigenetic landscape, affecting DNA methylation, histone modifications, and chromatin remodeling [82]. Such interactions are crucial for understanding the complex regulatory networks that control gene expression and maintain genome stability. By facilitating mutations, recombination, and genomic rearrangements, non-B DNA structures contribute to genetic diversity and evolutionary processes [6, 77, 82], essential for organisms’ adaptation to changing environments and developing new traits. These structures add layers of complexity and regulation beyond the canonical B-DNA form, enhancing phenotypic diversity and adaptability.

G-quadruplexes are four-stranded DNA structures formed by guanine-rich sequences commonly found in telomeres, promoter regions, and other genomic locations [84–86]. They play critical roles in regulating gene expression by influencing the binding of transcription factors. In telomeres, G-quadruplexes help protect chromosome ends from degradation and fusion, thereby maintaining genomic stability [87–89]. Their therapeutic potential is currently being explored in cancer treatment, as stabilizing G-quadruplexes can inhibit cancer cell proliferation [90–95]. Future research aims to identify G-quadruplex-forming sequences across different species and develop high-throughput methods to study their dynamics in vivo [96–98].

Cruciform DNA structures form when palindromic sequences create hairpin loops, resulting in a cross-shaped configuration [99–101]. These structures initiate DNA replication and regulate DNA repair processes by acting as signals for protein binding [99, 100, 102]. While cruciform structures can promote genetic diversity through genomic rearrangements, they can also potentially cause genomic instability [21]. Understanding this balance is crucial for insights into genome maintenance and evolution. Future research directions may include investigating the formation and resolution of cruciform DNA under various conditions and visualizing these structures in live cells.

Triplex DNA involves a third DNA strand binding to the major groove of a B-DNA duplex, forming a triple-stranded structure [103–105]. This can interfere with transcription factor binding, thereby regulating gene expression [106]. Triplex DNA also affects recombination and genomic rearrangements, contributing to genetic diversity and evolution [21]. Its therapeutic potential in gene editing and regulation is significant [21]. Its therapeutic potential in gene editing and regulation is substantial [104, 107–109], with future studies focusing on triplex DNA’s stability and formation conditions in different genomic contexts.

Technological advancements, such as single-molecule imaging, high-throughput sequencing, and CRISPR-based tools [110–115], enhance our ability to study non-B DNA structures. High-throughput sequencing technologies, such as ChIP-seq and ATAC-seq, can map the distribution of Z-DNA motifs and associated chromatin features across the genome [116–118]. These technologies allow for precise manipulation and observation of these structures in live cells, providing deeper insights into their functions. Targeting non-B DNA structures offers promising therapeutic avenues for treating various diseases, including cancer, genetic disorders, and neurodegenerative diseases. Drugs and molecules that specifically interact with these structures could modulate gene expression and genomic stability in a controlled manner.

Understanding the roles of various non-B DNA structures, including Z-DNA, provides a comprehensive view of the molecular mechanisms underlying genomic regulation, stability, and evolution. These structures play critical roles in gene expression, genome stability, and the generation of phenotypic diversity, highlighting their importance in the dynamic nature of genomes and organisms’ evolutionary processes. Continued research and technological advancements will uncover the diverse functions of non-B DNA structures and their potential applications in medicine and biotechnology.

## Conclusion

This study offers significant insights into the role of Z-DNA in avian genomes, particularly regarding its impact on genomic stability, gene regulation, and phenotypic diversity. Our analysis reveals that Z-DNA motifs are predominantly located in promoter regions, suggesting a potential role in regulating gene expression. The variation in Z-DNA density across different bird species indicates that these structures may contribute to species-specific regulatory mechanisms and evolutionary adaptations. We identified a negative correlation between Z-DNA density and developmental time in birds, suggesting that species with shorter developmental periods tend to have higher Z-DNA densities. This finding highlights the potential role of Z-DNA in influencing the timing and regulation of development in avian species.

Additionally, the association between Z-DNA density and traits such as body mass, egg mass, and genome size underscores the complex interactions between genome architecture and phenotypic characteristics. The enrichment of Z-DNA motifs in genes involved in nucleic acid binding, kinase activity, and translation regulation suggests that Z-DNA may play a crucial role in fine-tuning the expression of genes essential for maintaining cellular functions and responding to environmental changes. The potential for Z-DNA to induce genomic instability, leading to large-scale deletions or rearrangements, further underscores its role in facilitating phenotypic diversity and adaptive evolution.

Overall, this study highlights the importance of Z-DNA as a dynamic element in the avian genome, contributing to gene expression regulation, genomic stability maintenance, and the generation of phenotypic diversity. Future research should experimentally validate these associations and explore the specific molecular mechanisms through which Z-DNA influences avian biology. Understanding these dynamics will provide deeper insights into the evolutionary processes that shape bird genomes and their adaptation to diverse ecological niches.

## Supplementary Information

Supplementary Material 1.

Supplementary Material 2.

## Data Availability

All raw data were deposited in the figshare with DOI nos. 10.6084/m9.figshare.26925115 and 10.6084/m9.figshare.26924593.
