# Supplementary material for: A comprehensive study of Z-DNA density and its evolutionary implications in birds
Source: BMC Genomics. 2024 Nov 21;25:1123. doi: 10.1186/s12864-024-11039-x (PMC11580473; doi:10.1186/s12864-024-11039-x)
Supplement: Supplementary file 1 — Supplementary Material 1. [file 12864_2024_11039_MOESM1_ESM.pdf]

SUPPLEMENTARY TABLES

Table S1. Detail information of Z-DNA motifs predicted on nBMST. ZP1 in chickens and TG19 in Pigeon show high similarity to each other in length, composition and sequence.

**Chicken**				
Name	Length	Repeat	Sequence	Score
ZP1	40	TG	TGTGTGTGTGTGTGTGTGTGTGT GTGTGTGTGTGTGTGCG	80
ZP3	14	CA	CACACACACACACA	19
ZP6	25	TG	GTGCGTGTGCACGTGTGTGTGC ACA	91
ZP7	12	CA	CACGCACACACA	38
ZP4	22	TG	CACGTGTGTGTGTGCGTGTGCA	75

**Pigeon**				
Name	Length	Repeat	Sequence	Score
TG19	38	TG	GTGTGTGTGTGTGTGCGTGTGTG TGCGCACACACGTGT	121
1	19	CA	ACGCACGCACGCACACACA	93
2	17	CA	ACACACACACGCACACA	46
3	14	CA	ACACACACACACAC	19

4	20	TG	TGTGTGTGTGTGTGTGCGCA	61
5	12	CA	GCACACGCACGC	71

Table S2. Phylogenetic linear regression model fitting result of Z-DNA density and predictors.

Predictor	Model	All species			Non-Passeriformes			Passeriformes		
		**logLik**	**AIC**	**AICw**	**logLik**	**AIC**	**AICw**	**logLik**	**AIC**	**AICw**
Genome size	BM	-708.6	1423.3	0	-349.7	705.4	0.00087	-276.7	559.5	0
	OU	-649.7	1307.4	0.04109	-349.2	706.5	0.0005	-257.5	523.1	0.84553
	lambda	-646.5	1301.1	0.95891	-341.6	691.3	0.99863	-259.3	526.5	0.15447
Body mass	BM	-710.9	1427.9	0	-353.1	712.3	0	-283.0	571.9	0
	OU	-649.9	1307.9	0.99797	-349.2	706.5	0.00006	-265.5	539.0	0.96267
	lambda	-656.2	1320.3	0.00203	-339.4	686.9	0.99994	-268.7	545.5	0.03733
Egg mass	BM	-576	1158	0	-282.4	570.8	0.03269	-212.1	430.3	0
	OU	-520.7	1049.4	0.99894	-279.0	566.1	0.34276	-198.4	404.7	0.92414
	lambda	-527.5	1063.1	0.00106	-278.4	564.9	0.62455	-200.8	409.7	0.07586
Deve_T	BM	-602.8	1211.5	0	-290.6	587.2	0.17979	-230.8	467.7	0
	OU	-550	1108	0.99683	-290.2	588.3	0.12669	-214.4	436.9	0.8909
	lambda	-555.7	1119.5	0.00317	-288.4	584.9	0.69352	-216.6	441.1	0.1091
Incu_T	BM	-602.5	1211.0	0	-290	586	0.18214	-231.3	468.7	0
	OU	-548.1	1104.1	0.99842	-289	586	0.18214	-215.5	439.0	0.94268
	lambda	-554.5	1117.0	0.00158	-287.7	583.5	0.63572	-218.3	444.6	0.05732
Fled_T	BM	-602.8	1211.5	0	-291	588	0.20761	-230.0	466.1	0
	OU	-551.2	1110.4	0.99223	-290.7	589.4	0.1031	-213.6	435.3	0.86989

	lambda	-556.1	1120.1	0.00777	-288.8	585.6	0.68929	-215.6	439.1	0.13011
Deve_T (bodymass_adj)	BM	-600.6	1207.2	0	-290.5	587.0	0.03062	-228.7	463.4	0
	OU	-544.7	1097.4	0.99932	-288.4	584.7	0.0967	-215.2	438.4	0.95026
	lambda	-552	1112	0.00068	-286.2	580.3	0.87269	-218.2	444.3	0.04974
Incu_T (bodymass_adj)	BM	-601	1208	0	-290.1	586.1	0.04673	-229.5	465.0	0
	OU	-545.5	1098.9	0.99942	-288.3	584.5	0.104	-215.5	438.9	0.95026
	lambda	-552.9	1113.8	0.00058	-286.1	580.3	0.84927	-218.4	444.8	0.04974
Fled_T (bodymass_adj)	BM	-600.4	1206.8	0	-291.2	588.4	0.03243	-227.8	461.6	0
	OU	-544.4	1096.7	0.99913	-289.2	586.4	0.08817	-214.8	437.6	0.95026
	lambda	-551.4	1110.8	0.00087	-286.9	581.8	0.8794	-217.8	443.5	0.04974
Deve_T (eggmass_adj)	BM	-575.5	1157.0	0	-283.9	573.9	0.01131	-211.1	428.3	0
	OU	-519	1046	0.99877	-279.6	567.3	0.30652	-197.7	403.5	0.91293
	lambda	-525.7	1059.4	0.00123	-278.9	565.7	0.68217	-200.1	408.2	0.08707
Incu_T (eggmass_adj)	BM	-575.8	1157.6	0	-282.8	571.7	0.0201	-212.1	430.2	0
	OU	-520	1048	0.99909	-279.1	566.2	0.31437	-198.2	404.5	0.92056
	lambda	-527	1062	0.00091	-278.4	564.7	0.66553	-200.7	409.4	0.07944
Fled_T (eggmass_adj)	BM	-575.6	1157.2	0	-285	576	0.02859	-210.2	426.4	0
	OU	-519	1046	0.99797	-282.3	572.6	0.1565	-197.1	402.1	0.90887
	lambda	-525.2	1058.4	0.00203	-280.7	569.3	0.81491	-199.3	406.7	0.09112

Table S3. Phylogenetic linear regression model fitting result of STR density and predictors.

Predictor	Model	All species			Non-Passeriformes			Passeriformes		
		logLik	AIC	AICw	logLik	AIC	AICw	logLik	AIC	AICw
Genome size	BM	-973.1	1952.2	0	-624.2	1254.5	0	-340.3	686.6	0.00001
	OU	-951.3	1910.6	0	-604.3	1216.6	0.00003	-332.1	672.2	0.01799
	lambda	-932.8	1873.6	1	-593.9	1195.7	0.99997	-328.1	664.2	0.982
Body mass	BM	-967.7	1941.4	0	-624.3	1254.6	0	-337.2	680.4	0.00001
	OU	-945.7	1899.3	0	-604.4	1216.9	0.00003	-327.4	662.9	0.09112
	lambda	-928.7	1865.5	1	-594	1196	0.99997	-325.2	658.3	0.90886
Egg mass	BM	-777.5	1561.0	0	-522.6	1051.3	0	-251.4	508.9	0.00001
	OU	-757.1	1522.2	0	-506.3	1020.5	0.00015	-239.9	487.8	0.51249
	lambda	-743.8	1495.6	1	-497.4	1002.9	0.99985	-239.9	487.9	0.4875
Deve_T	BM	-812.6	1631.2	0	-535.2	1076.4	0	-273.0	552.1	0.00002
	OU	-791.8	1591.6	0	-517.7	1043.4	0.00014	-261.7	531.3	0.57443
	lambda	-777.7	1563.4	1	-508.8	1025.6	0.99986	-262.0	531.9	0.42555
Incu_T	BM	-812.3	1630.6	0	-535.3	1076.6	0	-272.1	550.2	0.00002
	OU	-791.9	1591.7	0	-518	1044	0.00012	-260.9	529.7	0.52497
	lambda	-777.9	1563.7	1	-509	1026	0.99988	-261.0	529.9	0.47501
Fled_T	BM	-812.5	1631.0	0	-535.1	1076.3	0	-273.3	552.5	0.00002
	OU	-791.7	1591.4	0	-517.6	1043.2	0.00014	-262.3	532.5	0.43781

	lambda	-777.7	1563.3	1	-508.8	1025.5	0.99986	-262	532	0.56217
Deve_T (bodymass_adj)	BM	-812.6	1631.2	0	-535.3	1076.7	0	-273.4	552.7	0.00003
	OU	-791.8	1591.6	0	-518.1	1044.2	0.00011	-263.1	534.1	0.3318
	lambda	-777.9	1563.7	1	-509	1026	0.99989	-262.3	532.7	0.66817
Incu_T (bodymass_adj)	BM	-812.6	1631.2	0	-535.3	1076.6	0	-273.4	552.7	0.00003
	OU	-791.8	1591.7	0	-518.1	1044.1	0.00011	-263.0	534.1	0.3318
	lambda	-777.9	1563.7	1	-508.9	1025.8	0.99989	-262.3	532.7	0.66817
Fled_T (bodymass_adj)	BM	-812.6	1631.1	0	-535.3	1076.6	0	-273.4	552.7	0.00003
	OU	-791.7	1591.4	0	-518.1	1044.2	0.00011	-263.1	534.1	0.3318
	lambda	-777.9	1563.7	1	-509	1026	0.99989	-262.3	532.7	0.66817
Deve_T (eggmass_adj)	BM	-777.5	1561.0	0	-522.6	1051.3	0	-251.3	508.5	0.00002
	OU	-757	1522	0	-506.2	1020.5	0.00015	-240.2	488.4	0.45016
	lambda	-743.8	1495.5	1	-497.4	1002.9	0.99985	-240	488	0.54982
Incu_T (eggmass_adj)	BM	-777.4	1560.9	0	-522.6	1051.2	0	-251.2	508.4	0.00002
	OU	-757.1	1522.2	0	-506.3	1020.5	0.00015	-240.1	488.3	0.46256
	lambda	-743.8	1495.6	1	-497.5	1002.9	0.99985	-240	488	0.53742
Fled_T (eggmass_adj)	BM	-777.5	1560.9	0	-522.6	1051.2	0	-251.3	508.6	0.00002
	OU	-756.9	1521.9	0	-506.2	1020.4	0.00016	-240.2	488.4	0.45016
	lambda	-743.7	1495.5	1	-497.4	1002.9	0.99984	-240	488	0.54982

Table S4. Phylogenetic linear regression model fitting result of genome size and developmental periods.

Predictor	Model	All species			Non-Passeriformes			Passeriformes		
		**logLik**	**AIC**	**AICw**	**logLik**	**AIC**	**AICw**	**logLik**	**AIC**	**AICw**
Deve_T	BM	-787.9	1581.9	0	-491.2	988.5	0	-287.9	581.9	0
	OU	-758.9	1525.8	0.00106	-479.6	967.3	0.00015	-273	554	0.6981
	lambda	-752.1	1512.1	0.99894	-470.9	949.7	0.99985	-273.8	555.7	0.3019
Incu_T	BM	-787	1580	0	-491.2	988.4	0	-287.3	580.7	0
	OU	-755.6	1519.2	0.00067	-476.9	961.8	0.00017	-273.6	555.2	0.82252
	lambda	-748.3	1504.6	0.99933	-468.2	944.4	0.99983	-275.1	558.2	0.17748
Fled_T	BM	-787.1	1580.2	0	-490.5	986.9	0	-287.2	580.4	0
	OU	-761.7	1531.5	0.00017	-480.4	968.9	0.0001	-272.4	552.8	0.67264
	lambda	-753.1	1514.2	0.99983	-471.2	950.4	0.9999	-273.1	554.2	0.32736
Deve_T	BM	-787.9	1581.9	0	-491.5	989.0	0	-287.2	580.5	0
(bodymass_adj)	OU	-761.1	1530.2	0.00007	-479.9	967.8	0.00013	-273	554	0.88471
	lambda	-751.6	1511.2	0.99993	-471	950	0.99987	-275.0	558.1	0.11529
Incu_T	BM	-788.1	1582.2	0	-491.2	988.3	0	-287.6	581.3	0
(bodymass_adj)	OU	-761.3	1530.6	0.0001	-480.1	968.2	0.00013	-273.3	554.6	0.87451
	lambda	-752.1	1512.2	0.9999	-471.1	950.3	0.99987	-275.2	558.5	0.12549
Fled_T	BM	-787.5	1581.0	0	-491.6	989.3	0	-286.7	579.4	0
(bodymass_adj)	OU	-761	1530	0.00005	-479.7	967.4	0.00013	-272.6	553.2	0.88848

	lambda	-751	1510	0.99995	-470.7	949.5	0.99987	-274.7	557.3	0.11152
Deve_T (eggmass_adj)	BM	-752.7	1511.4	0	-479.3	964.5	0	-264.8	535.6	0
	OU	-724.3	1456.6	0.0001	-466.5	941.1	0.00024	-250.3	508.6	0.81813
	lambda	-715.1	1438.2	0.9999	-458.2	924.5	0.99976	-251.8	511.6	0.18187
Incu_T (eggmass_adj)	BM	-753.6	1513.1	0	-479.9	965.8	0	-265.1	536.1	0
	OU	-724.4	1456.9	0.00027	-467.1	942.2	0.00032	-250.8	509.6	0.83061
	lambda	-716.2	1440.4	0.99973	-459.0	926.1	0.99968	-252.4	512.7	0.16939
Fled_T (eggmass_adj)	BM	-751.8	1509.5	0	-478.5	963.0	0	-264.3	534.6	0
	OU	-724.6	1457.2	0.0004	-466.3	940.7	0.00017	-249.7	507.4	0.77891
	lambda	-714.4	1436.7	0.9996	-457.6	923.3	0.99983	-250.9	509.9	0.22109

Table S6. Accession ID of genomes that were included in the analysis.

Species	accession ID	Species	accession ID	Species	accession ID
Acanthisitta chloris	GCA_016904835.1	*Cyanistes caeruleus*	GCF_002901205.1	*Nothoprocta perdicaria*	GCF_003342845.1
Accipiter gentilis	GCF_929443795.1	*Cyanocitta stelleri*	GCA_026167965.1	*Numida meleagris*	GCF_002078875.1
Acridotheres tristis	GCA_027559615.1	*Cygnus atratus*	GCF_013377495.2	*Nyctibius grandis*	GCA_013368605.1
Agelaius phoeniceus	GCF_020745825.1	*Cygnus olor*	GCF_009769625.2	*Oenanthe melanoleuca*	GCF_029582105.1
Alca torda	GCA_008658365.1	*Diglossa brunneiventris*	GCA_019023105.1	*Onychostruthus taczanowskii*	GCF_017590055.1
Alectoris rufa	GCA_019345075.1	*Dromaius novaehollandiae*	GCA_016128335.2	*Opisthocomus hoazin*	GCF_000692075.1
Amazona aestiva	GCA_017639355.1	*Dryobates pubescens*	GCF_014839835.1	*Oxyura jamaicensis*	GCF_011077185.1
Ammospiza caudacuta	GCF_027887145.1	*Egretta garzetta*	GCF_000687185.1	*Parus major*	GCF_001522545.3
Ammospiza nelsoni	GCF_027579445.1	*Empidonax traillii*	GCF_003031625.1	*Passer montanus*	GCF_014805655.1
Anas platyrhynchos	GCF_015476345.1	*Erithacus rubecula*	GCA_903797595.2	*Passerculus sandwichensis*	GCA_022577445.1
Anser cygnoides	GCA_013030995.1	*Falco biarmicus*	GCF_023638135.1	*Pavo muticus*	GCA_016647715.1
Apaloderma vittatum	GCF_000703405.1	*Falco cherrug*	GCF_023634085.1	*Pelecanus crispus*	GCA_030463565.1

Aphelocoma californica	GCA_028536675.1	*Falco naumanni*	GCF_017639655.2	*Pezoporus wallicus*	GCF_028554395.1
Aptenodytes forsteri	GCF_000699145.1	*Falco peregrinus*	GCF_023634155.1	*Phaethornis superciliosus*	GCA_023637945.1
Apteryx mantelli	GCF_001039765.1	*Falco rusticolus*	GCF_015220075.1	*Phasianus colchicus*	GCF_004143745.1
Apteryx rowi	GCF_003343035.1	*Ficedula albicollis*	GCF_000247815.1	*Phoenicopterus ruber*	GCA_009819775.1
Apus apus	GCF_020740795.1	*Fulmarus glacialis*	GCF_000690835.1	*Pipra filicauda*	GCF_003945595.2
Aquila chrysaetos	GCF_900496995.4	*Gallus gallus*	GCA_027408225.1	*Pluvialis apricaria*	GCA_017639485.1
Aratinga solstitialis	GCA_902168055.1	*Geospiza fortis*	GCF_000277835.1	*Podargus strigoides*	GCA_028020825.1
Artemisiospiza belli	GCA_021963965.1	*Geothlypis trichas*	GCA_009764595.1	*Poecile atricapillus*	GCF_030490865.1
Athene cunicularia	GCF_003259725.1	*Grus americana*	GCF_028858705.1	*Pogoniulus pusillus*	GCA_015220805.1
Aythya fuligula	GCF_009819795.1	*Gymnogyps californianus*	GCF_018139145.2	*Porphyrio hochstetteri*	GCA_020800305.1
Balearica regulorum	GCF_000709895.1	*Gypaetus barbatus*	GCA_028022735.1	*Pseudopodoces humilis*	GCF_000331425.1
Branta canadensis	GCA_006130075.1	*Haemorhous mexicanus*	GCA_027477595.1	*Pterocles gutturalis*	GCA_009769525.1
Bucorvus abyssinicus	GCA_009769605.1	*Haliaeetus albicilla*	GCA_947461875.1	*Pygoscelis adeliae*	GCF_000699105.1
Calidris pugnax	GCF_001431845.1	*Haliaeetus leucocephalus*	GCF_000737465.1	*Pyrgilauda ruficollis*	GCF_017590135.1
Callipepla	GCA_023055505.1	*Harpia harpyja*	GCF_026419915.1	*Rallus limicola*	GCA_022605955.1

californica					
Calypte anna	GCF_003957555.1	*Hemiprocne comata*	GCA_020745705.1	*Rhea americana*	GCA_003343005.1
Camarhynchus parvulus	GCA_902806625.1	*Hirundo rustica*	GCF_015227805.2	*Rhea pennata*	GCA_028389875.1
Caprimulgus europaeus	GCA_907165065.1	*Indicator indicator*	GCF_027791375.1	*Rhynochetos jubatus*	GCA_027574665.1
Cariama cristata	GCA_009819825.1	*Lagopus leucura*	GCF_019238085.1	*Rissa tridactyla*	GCA_028501385.1
Catharus ustulatus	GCF_009819885.2	*Lagopus muta*	GCF_023343835.1	*Serinus canaria*	GCF_022539315.1
Centrocercus urophasianus	GCF_019232065.1	*Laterallus jamaicensis*	GCA_022605575.1	*Setophaga petechia*	GCA_024362935.1
Chaetura pelagica	GCF_000747805.2	*Lepidothrix coronata*	GCF_001604755.1	*Spheniscus humboldti*	GCA_027474245.1
Chamaea fasciata	GCA_029207755.1	*Lonchura striata*	GCF_005870125.1	*Sterna hirundo*	GCA_009819605.1
Charadrius vociferus	GCF_000708025.1	*Lycocorax pyrrhopterus*	GCA_014706295.1	*Streptopelia turtur*	GCA_901699155.2
Chiroxiphia lanceolata	GCF_009829145.1	*Malurus melanocephalus*	GCF_030028575.1	*Strigops habroptila*	GCF_004027225.2
Chlamydotis macqueenii	GCF_000695195.1	*Manacus candei*	GCF_025592945.1	*Struthio camelus*	GCF_000698965.1
Chrysolophus pictus	GCA_003413605.1	*Manacus vitellinus*	GCF_001715985.3	*Sturnus vulgaris*	GCF_001447265.1
Ciconia maguari	GCA_017639555.1	*Melanerpes formicivorus*	GCA_026170545.1	*Sylvia atricapilla*	GCA_009819655.1

Colinus virginianus	GCA_008692595.2	*Meleagris gallopavo*	GCA_905368555.1	*Sylvia borin*	GCA_014839755.1
Coloeus monedula	GCA_013407035.1	*Melopsittacus undulatus*	GCF_000238935.1	*Taeniopygia guttata*	GCF_003957565.2
Columba livia	GCF_000337935.1	*Melospiza georgiana*	GCF_028018845.1	*Tauraco erythrolophus*	GCA_009769465.1
Corapipo altera	GCF_003945725.1	*Melospiza melodia*	GCA_022749695.1	*Theristicus caerulescens*	GCA_020745775.1
Corvus brachyrhynchos	GCF_000691975.1	*Melozone crissalis*	GCF_028551555.1	*Trogon surrucura*	GCA_020746105.1
Corvus cornix	GCF_000738735.6	*Merops nubicus*	GCA_009819595.1	*Tympanuchus cupido*	GCA_001870855.1
Corvus hawaiiensis	GCF_020740725.1	*Molothrus ater*	GCF_012460135.2	*Tyto alba*	GCF_018691265.1
Corvus kubaryi	GCF_017639235.1	*Motacilla alba*	GCF_015832195.1	*Vidua chalybeata*	GCA_026979565.1
Corvus moneduloides	GCF_009650955.1	*Myiopsitta monachus*	GCA_017639245.1	*Vidua macroura*	GCF_024509145.1
Coturnix japonica	GCF_001577835.2	*Myiozetetes cayanensis*	GCF_022539395.1	*Zonotrichia albicollis*	GCF_000385455.1
Crossoptilon mantchuricum	GCA_019593555.1	*Neopelma chrysocephalum*	GCF_003984885.1		
Cuculus canorus	GCF_017976375.1	*Nestor notabilis*	GCF_000696875.1		

Table S7. Whole genome Z-DNA density and STR density of 154 avian species.

Species	D_zdna	D_STR	Species	D_zdna	D_STR	Species	D_zdna	D_STR
Acanthisitta chloris	101.3787	539.7981	*Cyanistes caeruleus*	77.2479	631.5437	*Nothoprocta perdicaria*	134.8876	865.8691
Accipiter gentilis	106.5386	781.3964	*Cyanocitta stelleri*	88.69569	679.25	*Numida meleagris*	97.225	712.4837
Acridotheres tristis	84.41681	997.7168	*Cygnus atratus*	113.3116	1052.294	*Nyctibius grandis*	102.0341	631.2333
Agelaius phoeniceus	92.69496	728.2655	*Cygnus olor*	111.4434	1046.684	*Oenanthe melanoleuca*	96.57692	800.2058
Alca torda	114.5432	778.8669	*Diglossa brunneiventris*	97.01759	709.7463	*Onychostruthus taczanowskii*	90.94369	725.7709
Alectoris rufa	93.87379	833.4039	*Dromaius novaehollandiae*	136.8144	687.9952	*Opisthocomus hoazin*	100.2317	518.4233
Amazona aestiva	77.21239	522.0115	*Dryobates pubescens*	86.47563	828.4731	*Oxyura jamaicensis*	107.4374	1098.908
Ammospiza caudacuta	152.3073	716.8774	*Egretta garzetta*	100.4934	660.1934	*Parus major*	83.22941	655.5696

Ammospiza nelsoni	125.1958	708.2992	*Empidonax traillii*	92.75536	736.2411	*Passer montanus*	89.7625	671.6817
Anas platyrhynchos	117.9412	1125.187	*Erithacus rubecula*	90.62385	778.1633	*Passerculus sandwichensis*	144.5557	746.2974
Anser cygnoides	115.9811	1042.02	*Falco biarmicus*	108.307	688.8326	*Pavo muticus*	94.89057	694.4472
Apaloderma vittatum	91.82617	598.1486	*Falco cherrug*	102.658	663.4733	*Pelecanus crispus*	117.0511	754.8863
Aphelocoma californica	105.1785	672.5726	*Falco naumanni*	111.4016	715.6713	*Pezoporus wallicus*	96.34298	660.0649
Aptenodytes forsteri	104.552	636.4704	*Falco peregrinus*	112.7908	1230.03	*Phaethornis superciliosus*	84.83235	870.8873
Apteryx mantelli	113.3507	586.373	*Falco rusticolus*	109.6333	699.9942	*Phasianus colchicus*	96.67353	822.1176
Apteryx rowi	127.6911	684.4626	*Ficedula albicollis*	88.93482	773.0848	*Phoenicopter us ruber*	122.3864	675.2984
Apus apus	90.98	721.6	*Fulmarus glacialis*	101.9167	643.5798	*Pipra filicauda*	95.89722	658.4315
Aquila chrysaetos	105.2398	740.5545	*Gallus gallus*	102.2288	845.8766	*Pluvialis apricaria*	111.192	707.3552
Aratinga solstitialis	85.30684	584.206	*Geospiza fortis*	84.85047	607.5065	*Podargus strigoides*	139.1528	1353.887
Artemisiospiza	91.72214	808.79	*Geothlypis*	92.5037	678.0407	*Poecile*	124.9264	783.984

belli			*trichas*			*atricapillus*		
Athene cunicularia	92.19483	719.4371	*Grus americana*	120.0524	750.731	*Pogoniulus pusillus*	83.65827	666.5252
Aythya fuligula	112.4327	1176.881	*Gymnogyps californianus*	106.9274	708.0411	*Porphyrio hochstetteri*	109.9283	760.8748
Balearica regulorum	106.4168	681.646	*Gypaetus barbatus*	97.27299	863.1569	*Pseudopodoces humilis*	88.88077	696.8154
Branta canadensis	113.2252	1038.528	*Haemorhous mexicanus*	88.43043	750.4626	*Pterocles gutturalis*	103.1053	742.2779
Bucorvus abyssinicus	107.877	644.2602	*Haliaeetus albicilla*	100.8902	698.3818	*Pygoscelis adeliae*	101.2287	623.132
Calidris pugnax	106.0496	732.0878	*Haliaeetus leucocephalus*	97.17203	658.3568	*Pyrgilauda ruficollis*	89.63905	711.221
Callipepla californica	109.1642	924.3156	*Harpia harpyja*	95.65704	686.7444	*Rallus limicola*	107.2493	681.4457
Calypte anna	77.9934	807.4208	*Hemiprocne comata*	88.74224	654.3043	*Rhea americana*	124.7388	734.8052
Camarhynchus parvulus	100.9972	680.6774	*Hirundo rustica*	98.31081	819.9243	*Rhea pennata*	137.9937	847.4835
Caprimulgus europaeus	104.0771	755.3881	*Indicator indicator*	84.75043	924.8026	*Rhynochetos jubatus*	108.8662	634.518
Cariama	99.77049	687.632	*Lagopus leucura*	94.51471	800.3078	*Rissa*	113.0016	677.2213

cristata						*tridactyla*		
Catharus ustulatus	90.5531	700.8832	*Lagopus muta*	97.26117	858.8854	*Serinus canaria*	89.15714	719.2229
Centrocercus urophasianus	91.92871	782.4802	*Laterallus jamaicensis*	94.60144	595.7935	*Setophaga petechia*	94.30082	748.132
Chaetura pelagica	86.19375	659.2098	*Lepidothrix coronata*	91.2463	640.4926	*Spheniscus humboldti*	124.8912	717.314
Chamaea fasciata	92.41261	737.4193	*Lonchura striata*	83.33208	693.0274	*Sterna hirundo*	115.9439	746.1276
Charadrius vociferus	103.7492	689.0631	*Lycocorax pyrrhopterus*	100.3216	617.7505	*Streptopelia turtur*	107.5025	724.8831
Chiroxiphia lanceolata	94.78807	672.6083	*Malurus melanocephalus*	82.55392	677.2147	*Strigops habroptila*	90.15739	614.98
Chlamydotis macqueenii	98.11193	837.3284	*Manacus candei*	94.49018	682.7179	*Struthio camelus*	119.3057	659.7553
Chrysolophus pictus	93.87255	775.0843	*Manacus vitellinus*	87.45607	611.5037	*Sturnus vulgaris*	85.34519	669.1712
Ciconia maguari	107.5645	658.5806	*Melanerpes formicivorus*	84.18182	939.9114	*Sylvia atricapilla*	90.22243	747.5439
Colinus virginianus	107.5011	814.5916	*Meleagris gallopavo*	98.935	870.103	*Sylvia borin*	90.35905	738.0552
Coloeus	89.95385	620.2	*Melopsittacus*	76.72143	479.0607	*Taeniopygia*	85.98208	737.4849

monedula			*undulatus*			*guttata*		
Columba livia	108.9351	694.9982	*Melospiza georgiana*	133.519	694.4871	*Tauraco erythrolophus*	105.7792	686.908
Corapipo altera	90.87727	651.0636	*Melospiza melodia*	179.936	614.7478	*Theristicus caerulescens*	109.7333	700.6975
Corvus brachyrhynchos	85.10826	564.3651	*Melozone crissalis*	240.0794	714.4922	*Trogon surrucura*	103.5171	710.2291
Corvus cornix	91.47282	611.3359	*Merops nubicus*	91.0487	800.4652	*Tympanuchus cupido*	97.53061	790.4878
Corvus hawaiiensis	95.07565	653.3617	*Molothrus ater*	94.35963	711.3229	*Tyto alba*	101.7248	846.6256
Corvus kubaryi	88.59906	595.2925	*Motacilla alba*	94.1514	698.1981	*Vidua chalybeata*	90.05556	803.7102
Corvus moneduloides	91.57117	623.2523	*Myiopsitta monachus*	88.44188	641.8205	*Vidua macroura*	87.39286	799.0384
Coturnix japonica	96.95591	846.0323	*Myiozetetes cayanensis*	96.4287	714.3306	*Zonotrichia albicollis*	84.71238	622.9476
Crossoptilon mantchuricum	94.53069	816.2614	*Neopelma chrysocephalum*	95.00702	627.4298			
Cuculus canorus	90.79831	584.5449	*Nestor notabilis*	86.79333	558.68			

Table S8. Pairwise *p*-values of Z-DNA number in different regions upstream from a start codon.

	1 kb	2 kb	3 kb	4 kb	5 kb	6 kb	7 kb	8 kb	9 kb
2 kb	0.02372	-	-	-	-	-	-	-	-
3 kb	9.3e-14	1.2e-05	-	-	-	-	-	-	-
4 kb	< 2e-16	9.2e-14	9.3e-05	-	-	-	-	-	-
5 kb	< 2e-16	8.9e-14	5.3e-07	0.99420	-	-	-	-	-
6 kb	< 2e-16	< 2e-16	6.6e-14	0.00073	0.02947	-	-	-	-
7 kb	< 2e-16	< 2e-16	1.1e-13	4.1e-06	0.00050	0.98925	-	-	-
8 kb	< 2e-16	< 2e-16	7.6e-14	0.00200	0.06103	1.00000	0.95743	-	-
9 kb	< 2e-16	< 2e-16	7.8e-14	0.00234	0.06808	1.00000	0.94894	1.00000	-
10 kb	< 2e-16	< 2e-16	9.8e-14	2.6e-07	5.1e-05	0.88065	0.99998	0.75715	0.73347
