# Supplementary material for: A comprehensive study of Z-DNA density and its evolutionary implications in birds
Source: BMC Genomics. 2024 Nov 21;25:1123. doi: 10.1186/s12864-024-11039-x (PMC11580473; doi:10.1186/s12864-024-11039-x)
Supplement: Supplementary file 2 — Supplementary Material 2. [file 12864_2024_11039_MOESM2_ESM.pdf]

SUPPLEMENTARY FIGURES

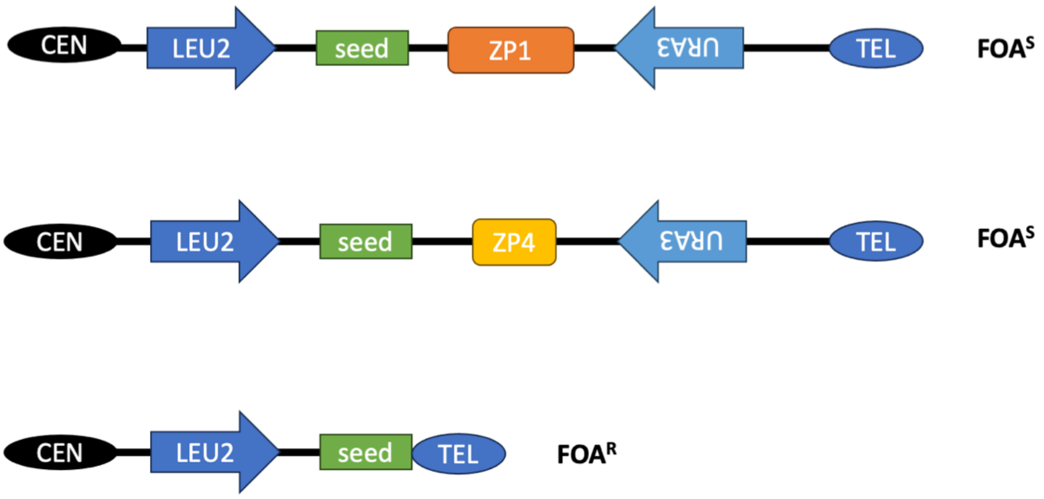

Figure S1. The ZP1 or ZP4 was integrated into a yeast artificial chromosome (YAC) between a telomere seed sequence $(G_4T_4)_{13}$ and the *URA3* gene (Top and middle panels). Breaks occurring within the fragile sequence undergo resection and telomere (TEL) addition, leading to the loss of the *URA3* gene and rendering the cells resistant to 5-FOA (5-FOA^R) (bottom panel). The YAC contains a *LEU2* marker gene for YAC maintenance and a centromere (CEN).

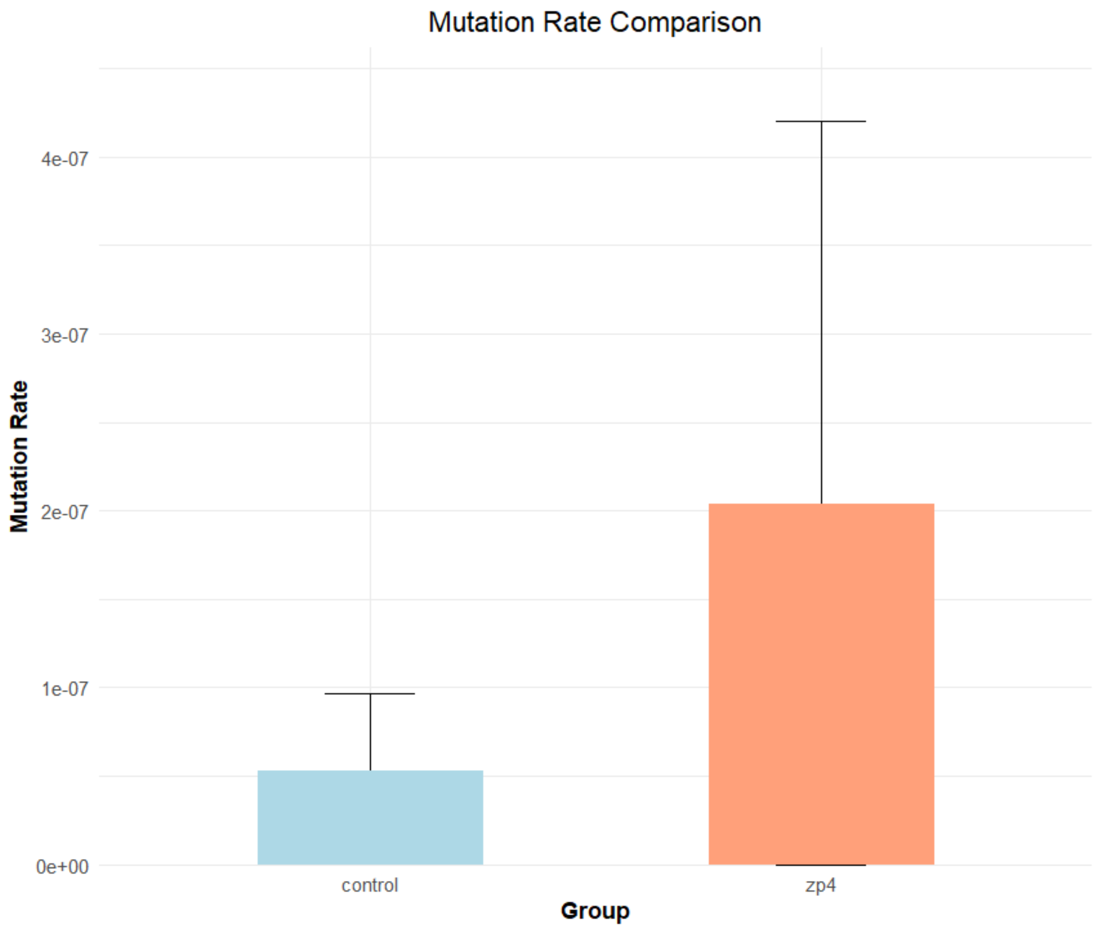

Figure S2. Fragility data of *S. cerevisiae* with the fragile sequence ZP4 integrated between the telomere seed sequence $(G_4T_4)_{13}$ and URA3 markers. YAC assays demonstrate that 5-FOA resistance increases in the presence of ZP4.

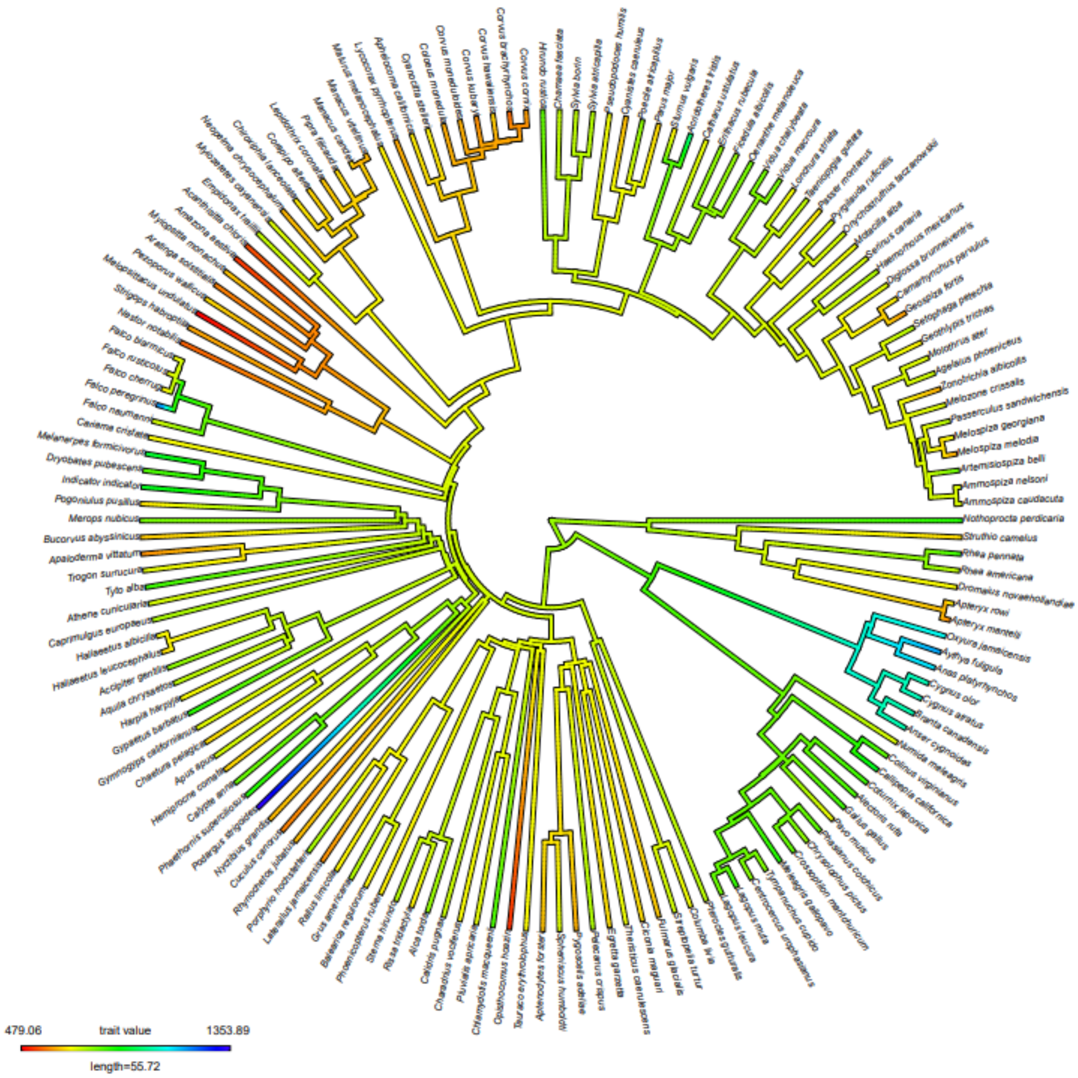

Figure S3. Ancestral state reconstruction of Short Tandem Repeat (STR) density across 154 avian species. The phylogenetic tree represents the evolutionary relationships among avian species, with branch colors indicating estimated STR density. The color gradient from blue to red reflects the variation in STR density, ranging from low (red) to high (blue) values, as indicated by the color scale at the bottom (479.06 to 1353.89 repeats per Mb).

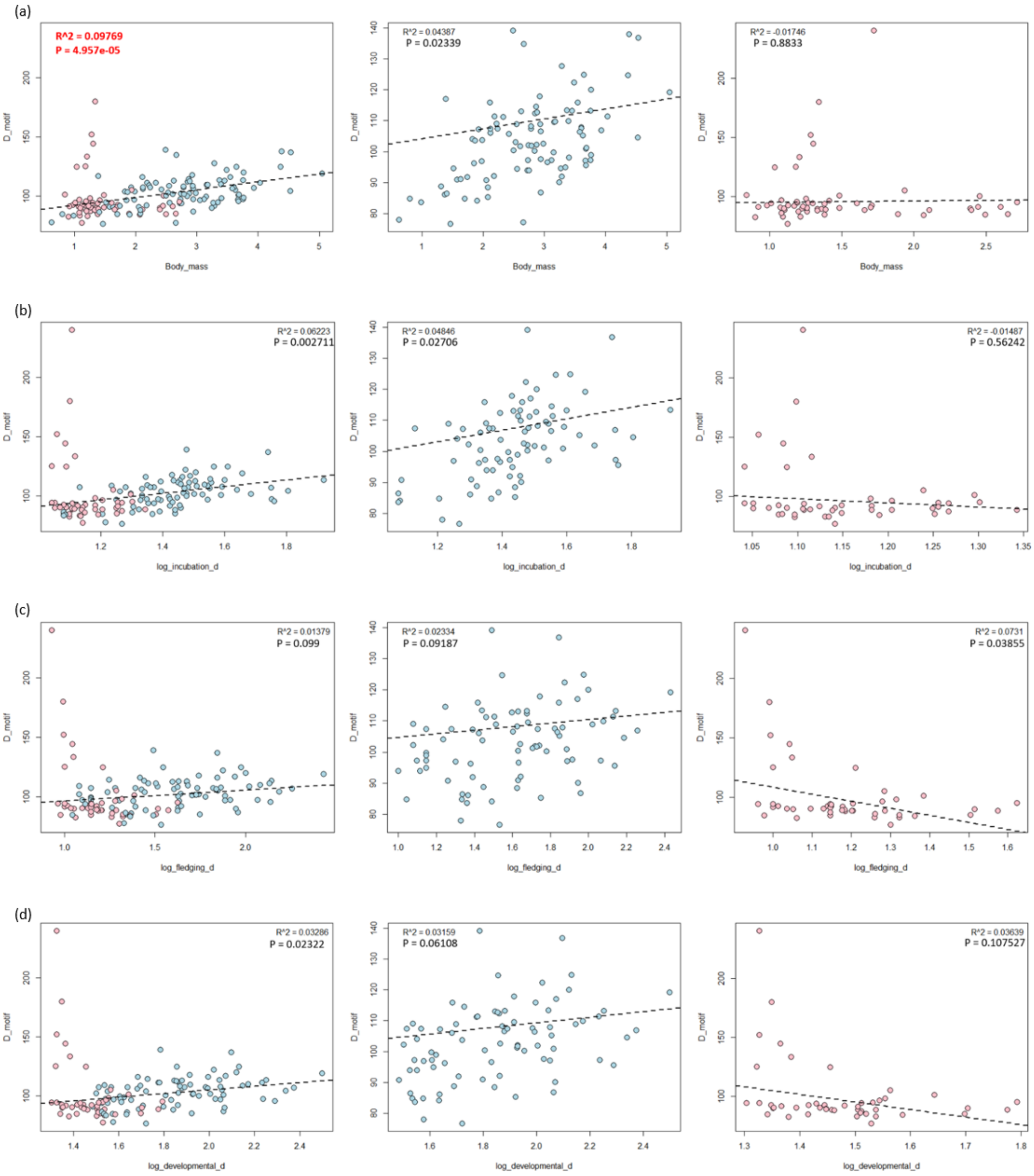

Figure S4. Phylogenetic linear regression analysis between Z-DNA density and key life history traits in avian species. The analysis includes: (a) adult body mass, (b) incubation duration, (c) fledging duration, and (d) total developmental time. Each column within the rows represents different bird groups: all birds (left), non-Passeriformes (middle), and Passeriformes (right). Each column within the rows represents different bird groups: all birds (left), non-Passeriformes (middle), and Passeriformes (right). Blue dots indicate non-Passeriformes species, while pink dots represent Passeriformes species. The R^2 values and p-values, provided in each plot, indicate the strength and significance of the relationships, respectively. The dashed lines represent the regression lines, illustrating the trend of the relationships between Z-DNA density and the corresponding traits. Values are red for weak correlations.

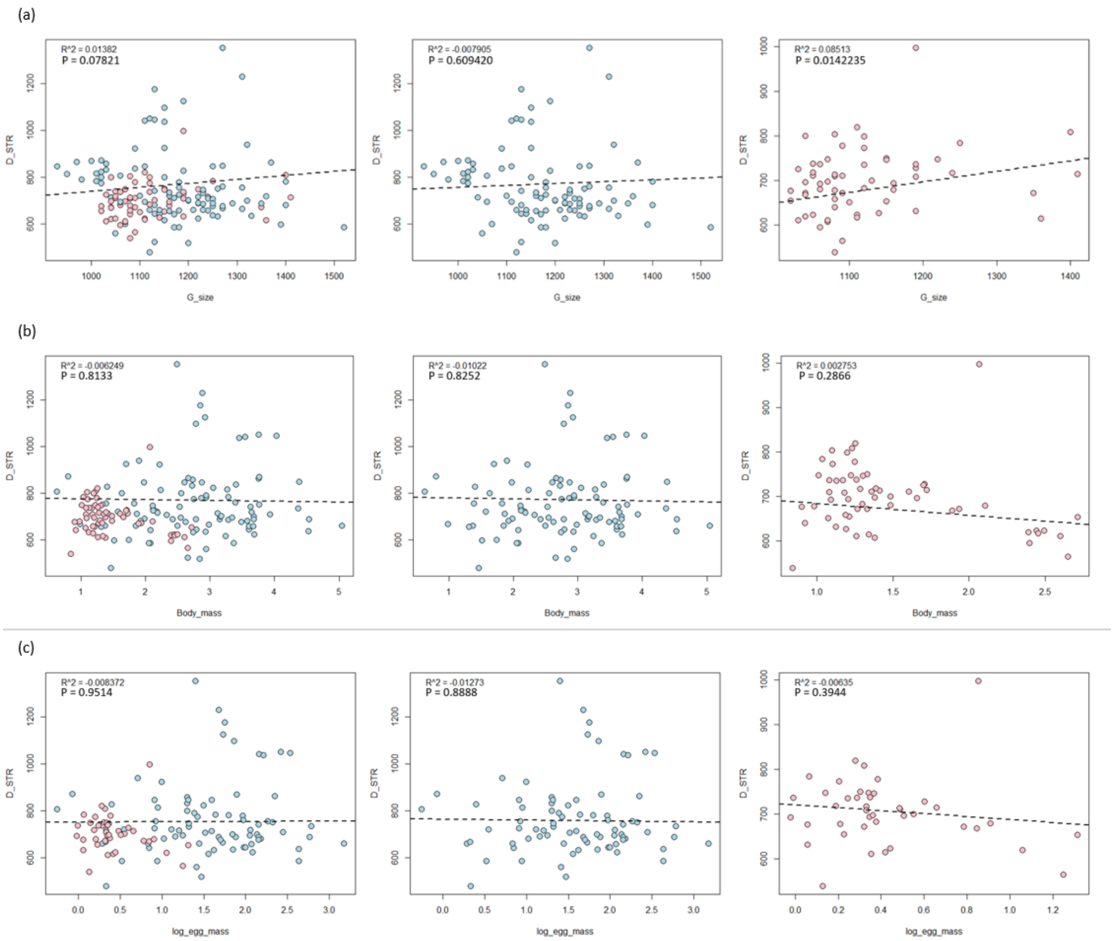

Figure S5. Phylogenetic linear regression between STR and (a) genome size, (b) adult body mass and (c) egg mass. Each column within the rows represents different bird groups: all birds (left), non-Passeriformes (middle), and Passeriformes (right). Blue dots indicate non-Passeriformes species, while pink dots represent Passeriformes species. The R^2 values and p-values, provided in each plot, indicate the strength and significance of the relationships, respectively. The dashed lines represent the regression lines, illustrating the trend of the relationships between Z-DNA density and the corresponding traits.

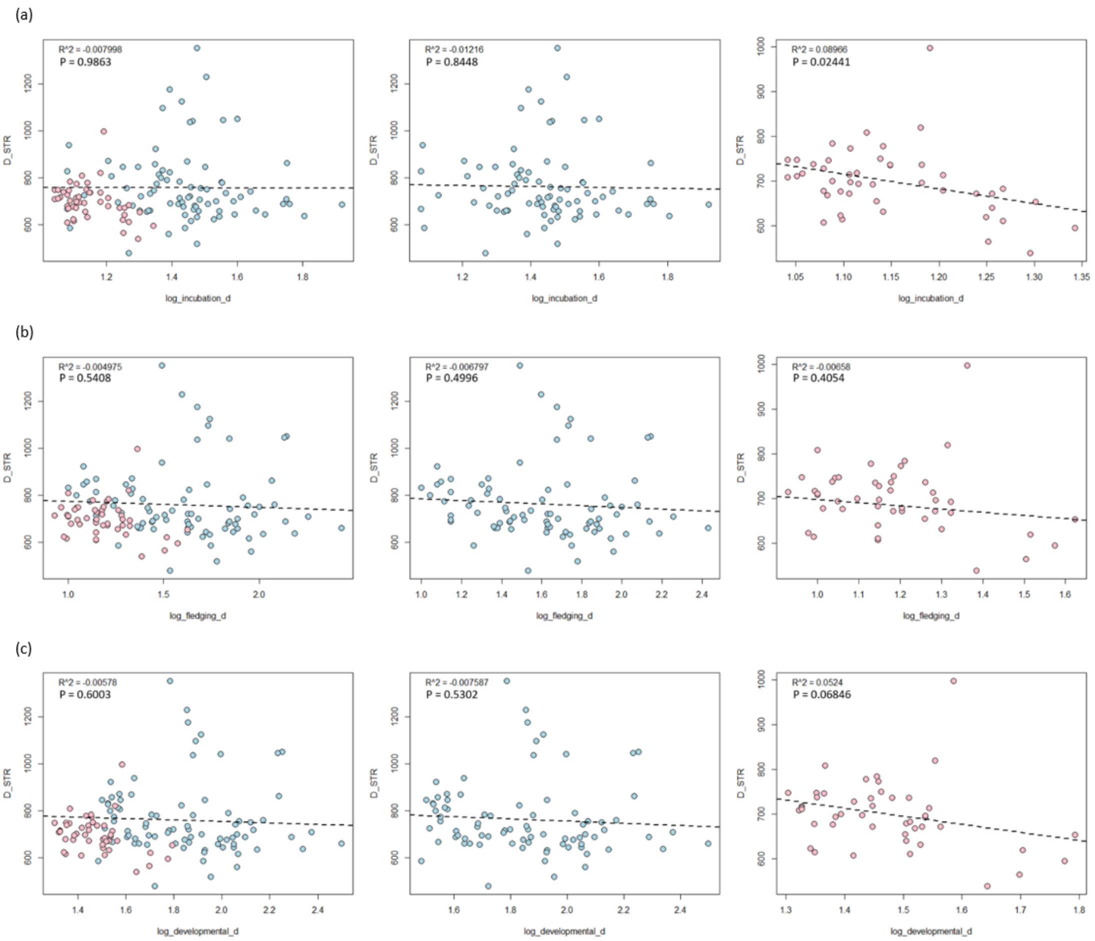

Figure S6. Phylogenetic linear regression between STR and developmental periods. (a) Incubation time. (b) Fledging time. (c) Developmental time. Each column within the rows represents different bird groups: all birds (left), non-Passeriformes (middle), and Passeriformes (right). Blue dots indicate non-Passeriformes species, while pink dots represent Passeriformes species. The R^2 values and p-values, provided in each plot, indicate the strength and significance of the relationships, respectively. The dashed lines represent the regression lines, illustrating the trend of the relationships between Z-DNA density and the corresponding traits.

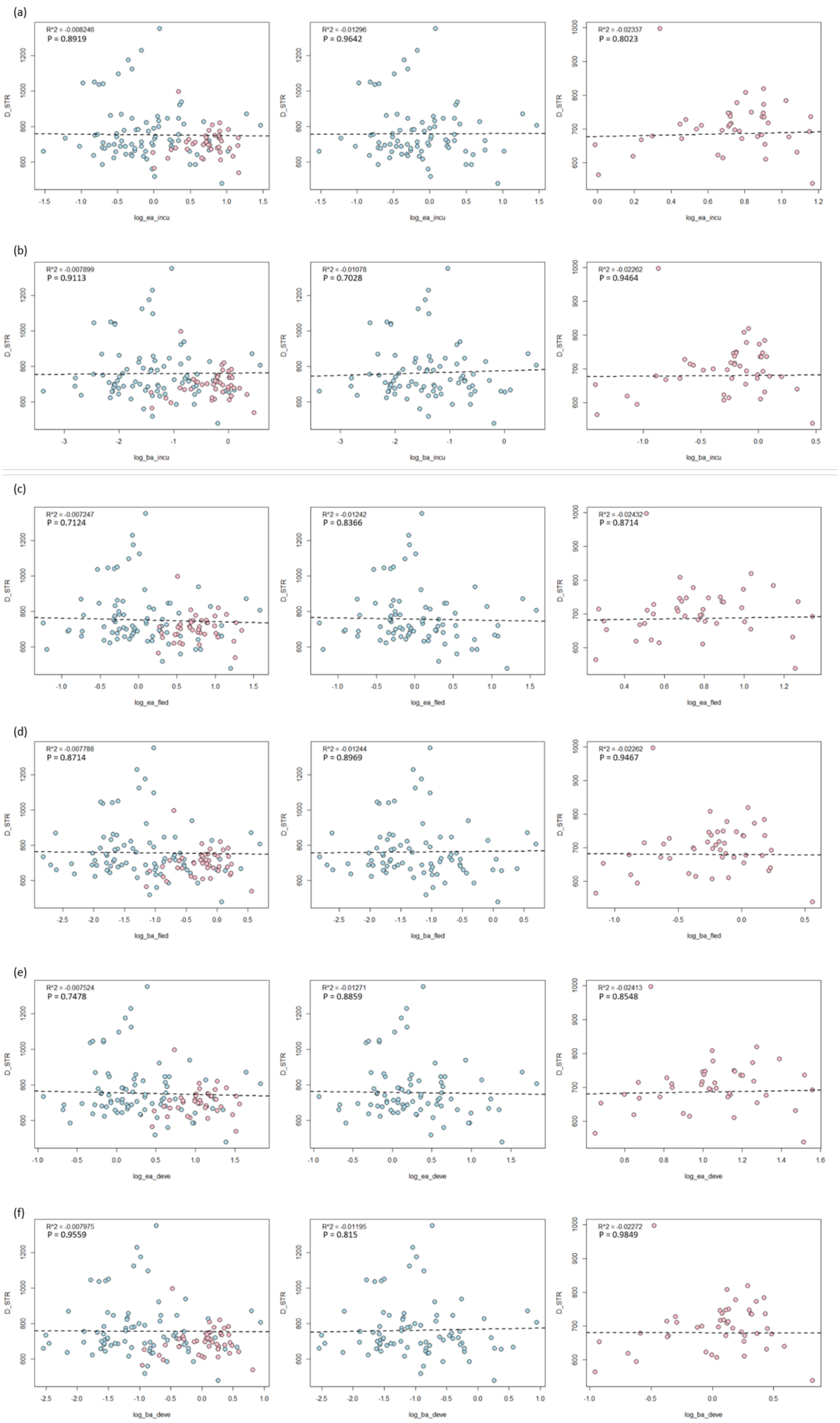
(a)
R^2 = -0.008246
P = 0.8919
R^2 = -0.01296
P = 0.9642
R^2 = -0.02337
P = 0.8023
D_STR
log_ea_incu
(b)
R^2 = -0.007899
P = 0.9113
R^2 = -0.01078
P = 0.7028
R^2 = -0.02262
P = 0.9464
D_STR
log_ba_incu
(c)
R^2 = -0.007247
P = 0.7124
R^2 = -0.01242
P = 0.8366
R^2 = -0.02432
P = 0.8714
D_STR
log_ea_fled
(d)
R^2 = -0.007788
P = 0.8714
R^2 = -0.01244
P = 0.8969
R^2 = -0.02262
P = 0.9467
D_STR
log_ba_fled
(e)
R^2 = -0.007524
P = 0.7478
R^2 = -0.01271
P = 0.8859
R^2 = -0.02413
P = 0.8548
D_STR
log_ea_deve
(f)
R^2 = -0.007975
P = 0.9559
R^2 = -0.01195
P = 0.815
R^2 = -0.02272
P = 0.9849
D_STR
log_ba_deve

Figure S7. Phylogenetic linear regression between STR and adjusted developmental periods. (a) Egg mass adjusted incubation time. (b) Adult body mass adjusted incubation time. (c) Egg mass adjusted fledging time. (d) Adult body mass adjusted fledging time. (e) Egg mass adjusted developmental time. (f) Adult body mass adjusted developmental time. The blue spots indicate the non-Passeriformes species, and the pink spots indicate the Passeriformes species.

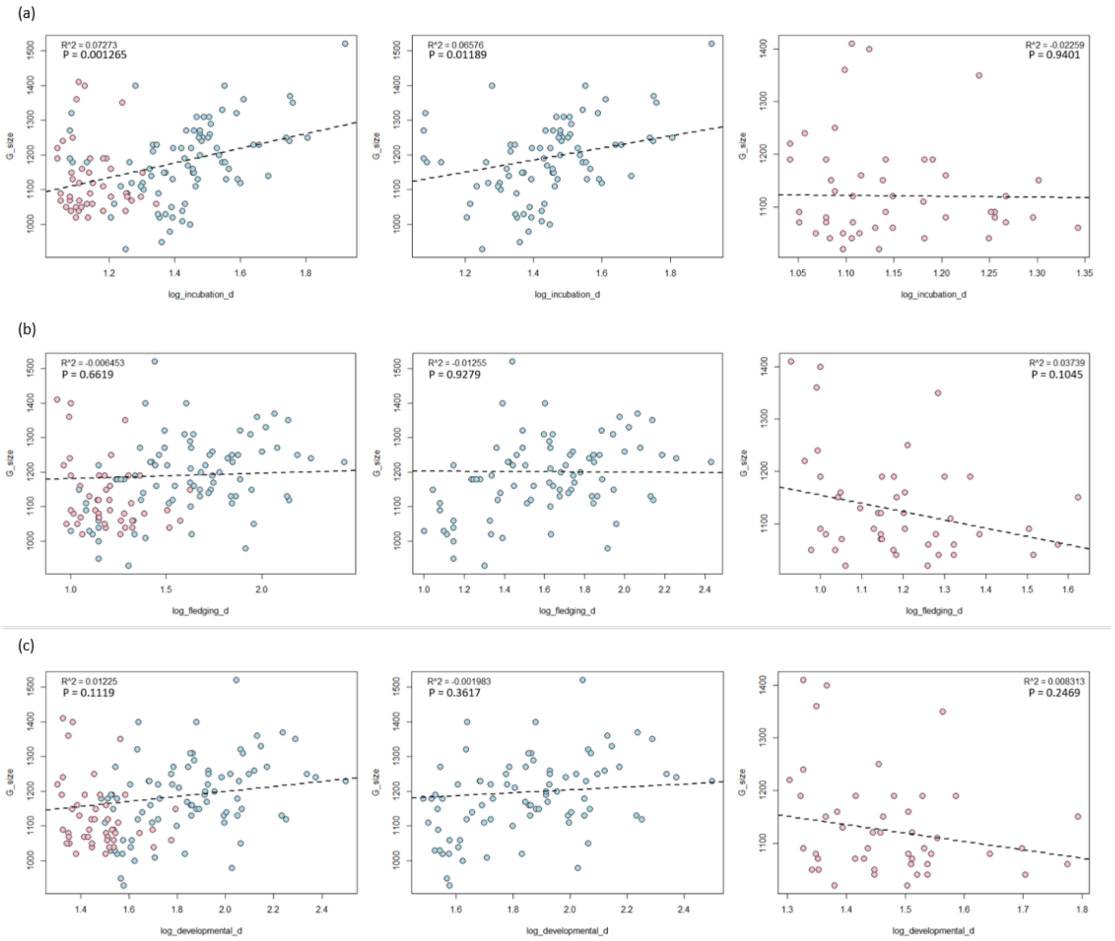

Figure S8. Phylogenetic linear regression between genome size and developmental periods. (a) Incubation time. (b) Fledging time. (c) Developmental time. Each column within the rows represents different bird groups: all birds (left), non-Passeriformes (middle), and Passeriformes (right). Blue dots indicate non-Passeriformes species, while pink dots represent Passeriformes species. The R^2 values and p-values, provided in each plot, indicate the strength and significance of the relationships, respectively. The dashed lines represent the regression lines, illustrating the trend of the relationships between Z-DNA density and the corresponding traits.

(a)

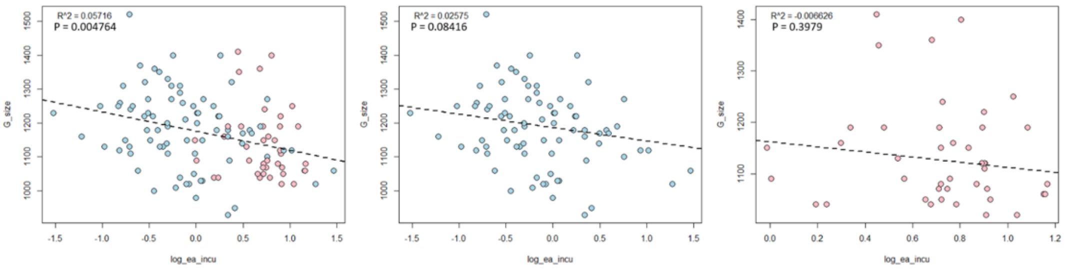
R^2 = 0.05716
P = 0.004764
R^2 = 0.02575
P = 0.08416
R^2 = -0.006626
P = 0.3979
G_size
log_ea_incu

(b)

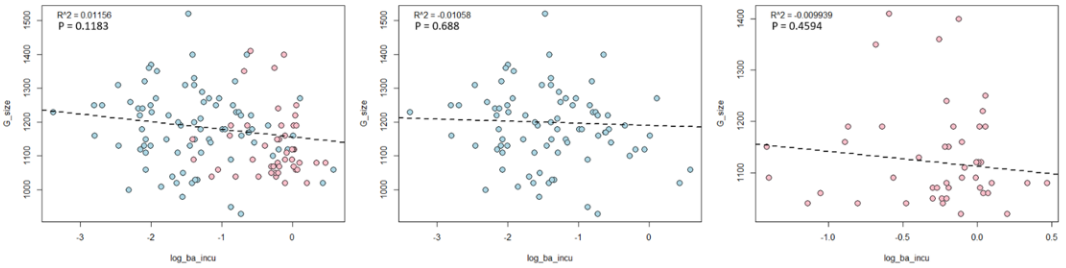
R^2 = 0.01156
P = 0.1183
R^2 = -0.01058
P = 0.688
R^2 = -0.009939
P = 0.4594
G_size
log_ba_incu

(c)

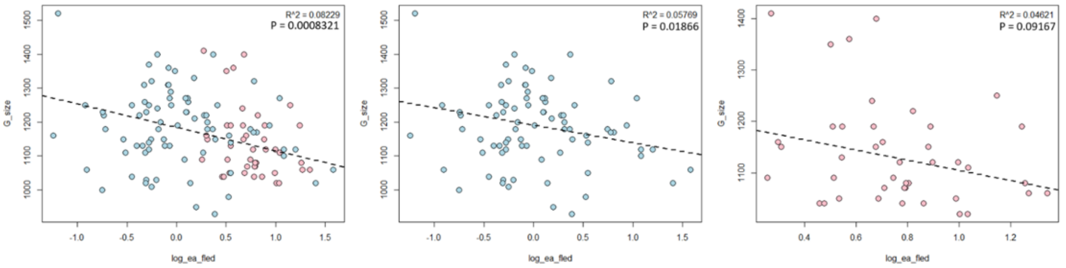
R^2 = 0.08229
P = 0.0008321
R^2 = 0.05769
P = 0.01866
R^2 = 0.04621
P = 0.09167
G_size
log_ea_fled

(d)

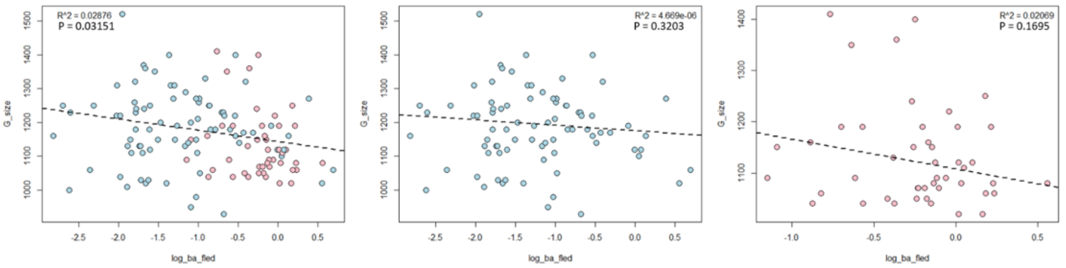
R^2 = 0.02876
P = 0.03151
R^2 = 4.669e-06
P = 0.3203
R^2 = 0.02069
P = 0.1695
G_size
log_ba_fled

(e)

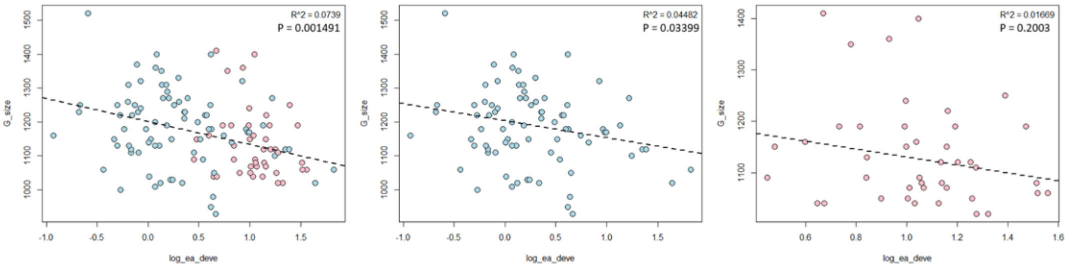
R^2 = 0.0739
P = 0.001491
R^2 = 0.04482
P = 0.03399
R^2 = 0.01669
P = 0.2003
G_size
log_ea_deve

(f)

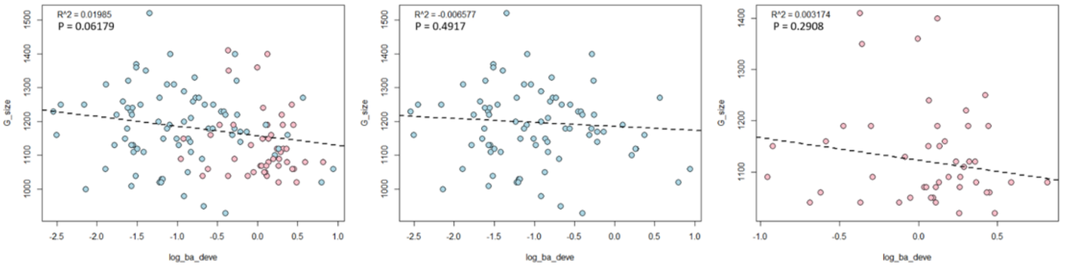
R^2 = 0.01985
P = 0.06179
R^2 = -0.006577
P = 0.4917
R^2 = 0.003174
P = 0.2908
G_size
log_ba_deve

Figure S9. Phylogenetic linear regression between genome size and adjusted developmental periods. (a) Egg mass adjusted incubation time. (b) Adult body mass adjusted incubation time. (c) Egg mass adjusted fledging time. (d) Adult body mass adjusted fledging time. (e) Egg mass adjusted developmental time. (f) Adult body mass adjusted developmental time. Each column within the rows represents different bird groups: all birds (left), non-Passeriformes (middle), and Passeriformes (right). Blue dots indicate non-Passeriformes species, while pink dots represent Passeriformes species. The R^2 values and *p*-values, provided in each plot, indicate the strength and significance of the relationships, respectively. The dashed lines represent the regression lines, illustrating the trend of the relationships between Z-DNA density and the corresponding traits.

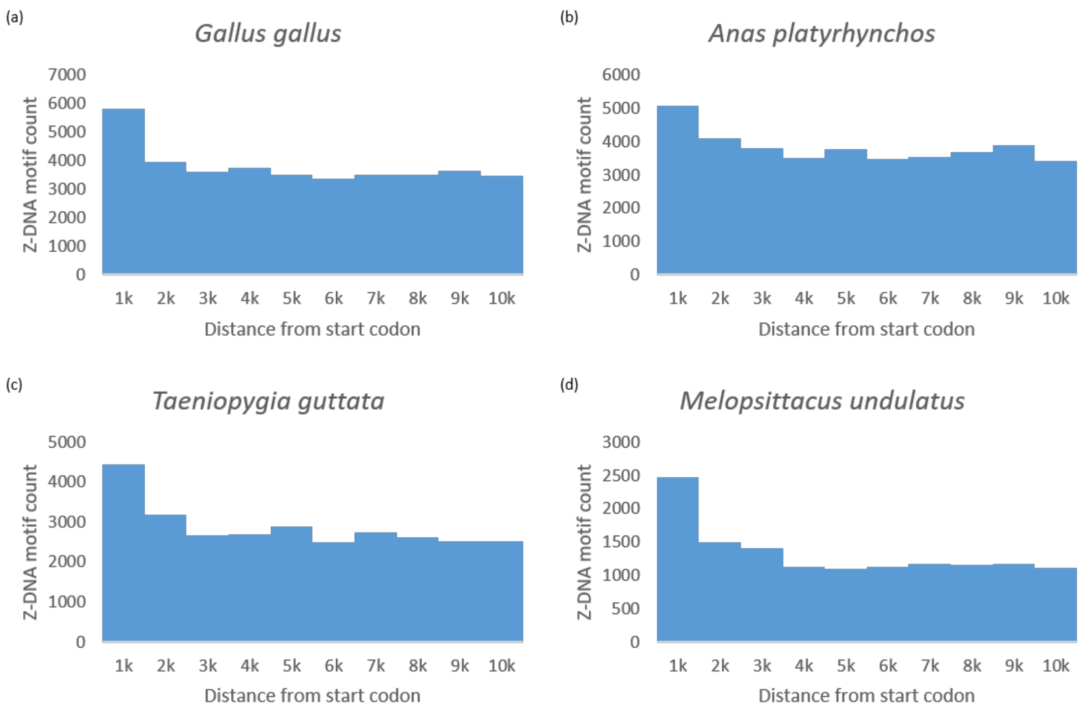

Figure S10. Z-DNA distribution in the upstream of start codons in (a) chicken, (b) duck, (c) zebra finch, and (d) budgerigar.

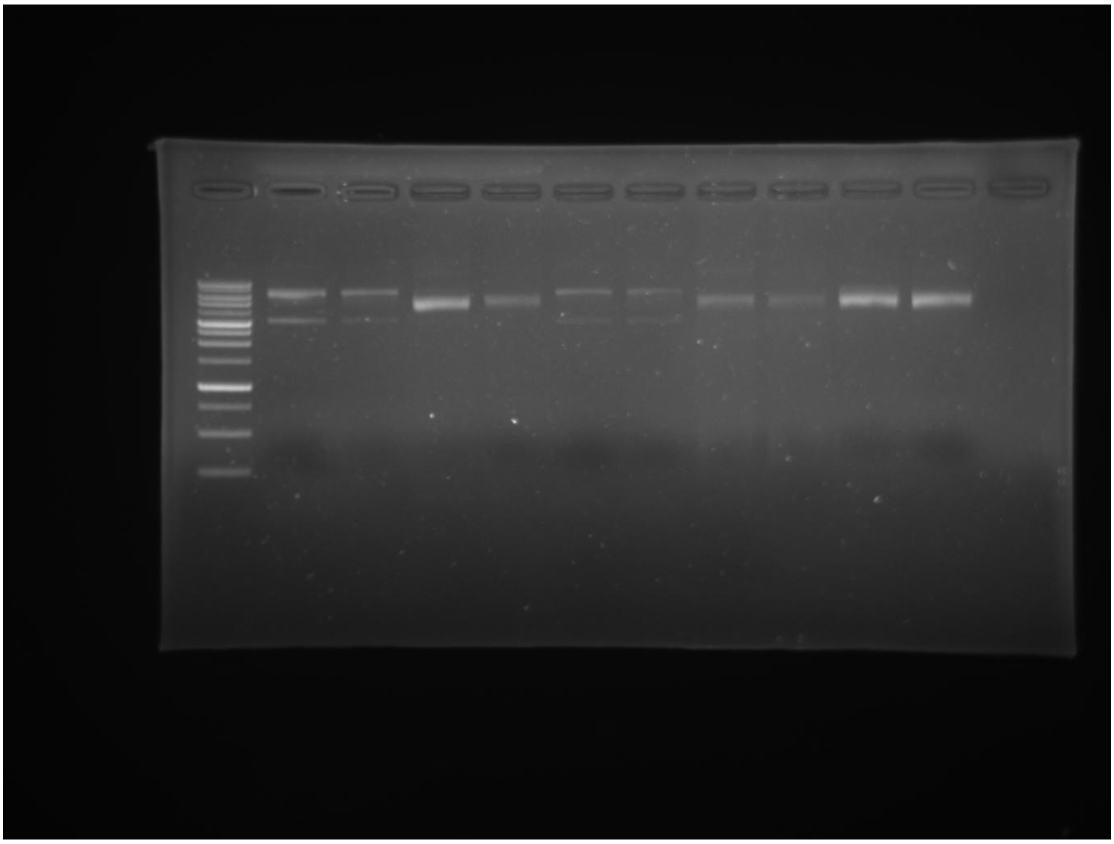

Figure S11. Original gel image of Fig. 1b. An S1 nuclease digestion assay was performed on pUC57 vectors with and without Z-DNA insertions.
